# Epigenetic Activation of ANGPTL4 Drives EBV‐Negative Gastric Cancer via Neutrophil Recruitment and NET Release via Stabilizing ANXA5

**DOI:** 10.1002/advs.78595

**Published:** 2026-11-05

**Authors:** Dandan Li, Xinqi Li, Xiangyin Luo, Jiayi Wang, Yiwei Zha, Zeng Zhou, Yanyan Chen, Qi Wang, Lingyun Xia, Weidong Leng, Shanshan Qin

**Affiliations:** ^1^ Department of Stomatology Hubei Provincial Clinical Research Center For Umbilical Cord Blood Hematopoietic Stem Cells Taihe Hospital Hubei University of Medicine Shiyan Hubei China; ^2^ Hubei Key Laboratory of Embryonic Stem Cell Research School of Basic Medical Sciences Hubei University of Medicine Shiyan Hubei China; ^3^ Shiyan Key Laboratory of Comprehensive Prevention and Treatment of Oral Cancer Academy of Bio‐Medicine Research Hubei University of Medicine Shiyan China

**Keywords:** DNA methylation, epithelial‐mesenchymal transition, Epstein‐Barr virus, immunosuppressive microenvironment, neutrophils

## Abstract

EBV‐positive (EBVaGC) and EBV‐negative (EBVnGC) gastric cancers exhibit distinct clinical outcomes, prognostic features, and immune infiltration landscapes; however, the molecular mechanisms driving these subtype differences, as well as the subtype‐specific epigenetic regulation and biological functions of ANGPTL4 within the immune microenvironment, remain unclear. Here, through integrated analysis of multi‐omics data, we discovered that EBV infection induces marked hypermethylation at the ANGPTL4 promoter, leading to transcriptional silencing of ANGPTL4 in EBVaGC. Conversely, hypomethylation‐mediated ANGPTL4 overexpression is clinically associated with poor prognosis and distant metastasis in EBVnGC. Mechanistically, the C‐terminal secreted form (cANGPTL4) promotes GC progression by enhancing proliferation, epithelial‐mesenchymal transition (EMT), and neutrophil recruitment. Clinically, ANGPTL4 expression is positively correlated with neutrophil infiltration and neutrophil extracellular traps (NETs) levels in GC. Spatial transcriptomics and immunohistochemistry (IHC) analyses confirmed the spatial colocalization of ANGPTL4 with tumor‐infiltrating neutrophils. cANGPTL4 mediates tumor‐associated neutrophil (TAN) recruitment via the NF‐κB/CXCL8 axis and directly binds and stabilizes neutrophil‐derived ANXA5 to facilitate NET release, thereby driving GC progression. In conclusion, ANGPTL4 represents a potential intervention target for EBVnGC. Targeting ANGPTL4 may exert dual anti‐tumor effects by directly suppressing GC proliferation, EMT, and metastasis, and by remodeling the immunosuppressive microenvironment via attenuating TANs infiltration and NET release.

## Graphical abstract

ANGPTL4 exhibits distinct promoter methylation patterns and expression profiles in EBV‐positive versus EBV‐negative gastric cancer. Secreted C‐terminal ANGPTL4 (cANGPTL4) acts as the dominant functional isoform driving GC proliferation, EMT, and neutrophil infiltration. cANGPTL4 promotes neutrophil recruitment via NF‐κB/CXCL8 axis. cANGPTL4 drives GC progression by inducing NETs release via directly stabilizing ANXA5.

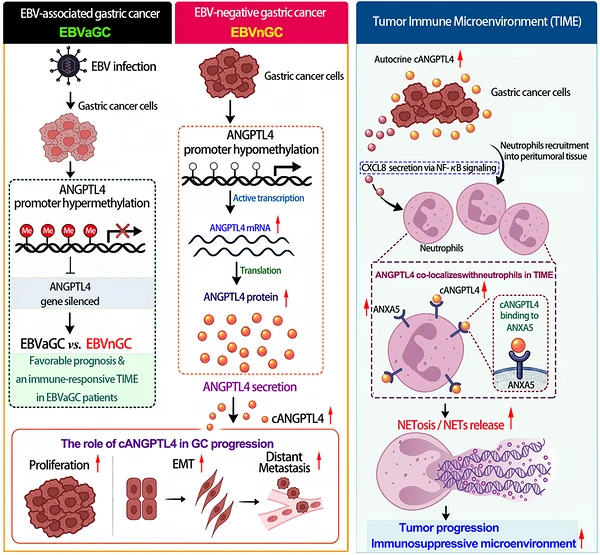

## 1 Introduction

Gastric cancer (GC) is one of the most common malignancies worldwide, characterized by high morbidity and mortality rates [1, 2, 3]. Annually, it accounts for nearly 1 million new cases and over 650,000 deaths, posing a pressing global public health challenge [4]. The incidence and mortality of GC vary significantly by region—with the highest rates in East Asia compared to North America and Northern Europe [5]. And the global burden of GC is projected to escalate further by 2040 [6, 7, 8]. Furthermore, mirroring trends observed in other gastrointestinal malignancies, the incidence of early‐onset GC has been rising steadily over the past decade, presenting new clinical challenges [9].

Epstein‐Barr virus (EBV), a member of the Herpesviridae family, infects over 90% of adults worldwide and typically establishes latency in B lymphocytes. EBV infection is closely associated with the initiation and progression of various malignancies, including nasopharyngeal carcinoma, stomach cancer, and lymphomas [10]. GC, originating from the gastric mucosal epithelium, is a highly heterogeneous tumor that can occur in various regions such as the cardia, fundus, body, and pylorus [11, 12, 13]. In 2014, The Cancer Genome Atlas (TCGA) project identified four distinct molecular subtypes of GC: Epstein‐Barr virus‐positive (EBV), microsatellite instability (MSI), chromosomal instability (CIN), and genomically stable (GS) [14]. Among these, Epstein‐Barr virus‐associated gastric cancer (EBVaGC) is a distinct molecular subtype of GC, characterized by clonal EBV infection within tumor cells. The gold standard for the definitive diagnosis of EBVaGC is EBER (EBV‐encoded small RNA) in situ hybridization (EBER‐ISH) [15]. Accounting for approximately 9% to 10% of all GC cases, EBVaGC exhibits distinct clinicopathological features, including a male predominance, a predilection for the proximal stomach (such as the cardia and fundus), and a frequent lymphoepithelioma‐like histology with dense lymphocytic infiltration [16]. At the molecular level, EBVaGC is driven by unique oncogenic mechanisms, frequently harboring PIK3CA mutations, global CpG island hypermethylation, and frequent amplification of PD‐L1 [17]. Additionally, EBVaGC exhibits a unique immune microenvironment characterized by abundant CD8^+^ T cell infiltration and high PD‐L1 expression, conferring an excellent clinical prognosis and robust immunotherapeutic response [18, 19, 20].

The angiopoietin‐like protein (ANGPTL) family comprises secreted proteins structurally similar to angiopoietins, which play crucial roles in regulating angiogenesis, lipid metabolism, and inflammatory responses [21]. Among them, ANGPTL4 (angiopoietin‐like 4) has attracted considerable attention due to its unique biological functions in cancer and other diseases [22]. ANGPTL4 is a secreted protein consisting of a signal peptide, an N‐terminal coiled‐coil domain, and a C‐terminal fibrinogen‐like domain. The N‐terminal fragment primarily participates in lipid metabolism by inhibiting the activity of lipoprotein lipase (LPL), whereas the C‐terminal fragment regulates cell adhesion, migration, and vascular permeability by binding to corresponding receptors [23].

The functional roles of ANGPTL4 in EBVaGC and EBV‐negative gastric cancer (EBVnGC) have not been reported yet. Besides, it remains controversial whether ANGPTL4 functions as a tumor suppressor or an oncogene in GC. In 2014, Takada and colleagues found that ANGPTL4 functioned as a tumor suppressor gene in GC by inhibiting angiogenesis [24]. However, a study by Zhen et al. demonstrated that ANGPTL4 exerts an oncogenic role in gastric cancer (GC), and its T266M mutation reduces the binding affinity for integrin α5β1, thereby suppressing GC progression [25]. Liu and colleagues found that COL1A1‐positive endothelial cells promote GC progression by inducing endothelial‐to‐mesenchymal transition via the ANGPTL4‐SDC4 axis [26]. In this study, we found that secreted ANGPTL4 (cANGPTL4) drives GC progression through a dual mechanism: it directly promotes tumor cell proliferation, EMT, and metastasis, while simultaneously orchestrating the tumor microenvironment. Specifically, cANGPTL4 activates the NF‐κB/CXCL8 pathway to recruit TANs and interacts with neutrophil‐derived ANXA5 to induce NETs formation. Thus, targeting cANGPTL4 may achieve a dual anti‐tumor effect by suppressing both tumor progression and neutrophil‐mediated immunosuppression.

## 2 Materials and Method

### 2.1 Sample Collection

All patients enrolled in this study were given the informed consent before inclusion. The stomach cancer cohort used in this study was obtained from GC patients with informed consent in the Taihe Hospital of Hubei University of Medicine (Shiyan, Hubei, China). All the patients enrolled in this study provided written informed consent. All samples were pathologically confirmed. This study was approved by the Human Research Ethics Committee of Hubei University of Medicine.

### 2.2 Data Acquisition

Gene expression profile data and corresponding clinical information of the TCGA stomach adenocarcinoma (STAD) cohort were obtained from the cBioPortal database [27]. Bulk transcriptomic data (GSE62254, GSE51575, GSE135644, GSE240267, GSE122401, and GSE184336) used in this study were obtained from the Gene Expression Omnibus (GEO) database. Survival analysis was performed using the survival R package (version 3.3.1). The survminer R package (version 0.4.9) was used to select the best cutoff values between the lower (Low group) and upper quartiles (High group) to acquire the best performing threshold of survival curves. The ggplot2 R package (version 3.4.4) was used to visualize the results of survival curves.

### 2.3 Spatial Transcriptomics Data Analysis

Spatial transcriptomics data analysis was performed using the Seurat package (v5.1) in R (version 4.4.2). The spatial transcriptomics dataset of gastric cancer (GSE251950) was obtained from the GEO database [28]. The filtered expression matrix was loaded using the “Load10X_Spatial()” function to construct a Seurat object containing both spot‐level gene expression data and the associated histological image. Normalization was conducted using the “SCTransform()” function, which applies a regularized negative binomial regression model to remove technical noise while preserving biological heterogeneity. Finally, the spatial distribution of gene expression was visualized using the “SpatialFeaturePlot()” function.

### 2.4 DNA Methylation Analysis

The DNA methylation level of each CpG site in the promoter of the ANGPTL4 gene was determined by the normalized β value. The DNA methylation data (β value) in GC cohorts (GSE99111, GSE30601, and GSE85464) or gastric cell lines (GSE89269) were downloaded from the GEO database. The DNA methylation data and gene expression data in different subtypes of GC in TCGA_STAD cohort were analyzed using the LinkedOmics web tool.

### 2.5 Cell Culture and Treatment

The human GC cell lines AGS (RRID: CVCL_0139) and HGC‐27 (RRID: CVCL_1279) and the normal gastric epithelial cell line GES‐1 (RRID: CVCL_EQ22) were obtained from the Shanghai Cell Bank. GES‐1 cells were cultured in DMEM medium supplemented with 10% fetal bovine serum (FBS; RYS‐KF22‐05, Rongye, Lanzhou, China) and 1% penicillin‐streptomycin solution. HGC‐27 and AGS cells were cultured in RPMI‐1640 medium supplemented with 10% FBS and 1% penicillin‐streptomycin solution. All cells were maintained in a humidified incubator at 37°C with 5% CO_2_. GC cells were treated with recombinant human cANGPTL4 protein (100 ng mL^−1^, Cat. No.: CW53, Novoprotein, Shanghai, China) for 72 h, with a vehicle control group set up in parallel.

HGC‐27 cells were transduced with ANGPTL4‐overexpressing lentivirus as described above. Transduced cells were seeded into T25 culture flasks and cultured for 48 h. The conditioned medium (CM) was collected and centrifuged at 2,000 rpm for 5 min to remove cellular debris, and the supernatant was stored at 4°C until use. Wild‐type HGC‐27 cells were seeded into 6‐well plates and cultured until reaching approximately 20% confluence. The culture medium was then replaced with 1 mL of the conditioned medium, and the cells were incubated at 37°C with 5% CO_2_. The conditioned medium was replaced with 1 mL of fresh ANGPTL4‐overexpressing conditioned medium every 24 h. After 72 h of treatment, the cells were harvested for subsequent analysis.

### 2.6 Lentiviral Transduction and Establishment of Stable Cell Lines

Stable cell models overexpressing full‐length ANGPTL4 protein were successfully established in HGC‐27 and AGS cell lines using lentiviral transduction (Genechem, Shanghai, China). Briefly, AGS and HGC‐27 cells in the logarithmic growth phase were harvested, counted, and seeded into 6‐well plates at 2 × 10^5^ cells per well in 2.5 mL of complete medium, with the plating density controlled to reach 30%–50% confluence. After incubation for at least 6 h to allow complete attachment, the medium was removed and cells were washed once with PBS. Fresh complete medium (1.0 mL per well) was added. Lentiviruses carrying a puromycin resistance gene were thawed on ice, and the optimal multiplicity of infection (MOI) for each cell line was added together with 20 µL of transduction enhancer. Cells were incubated at 37°C with 5% CO_2_, and the medium was replaced with fresh complete medium after 24 h. At 48 h post‐transduction, fluorescence expression was examined under an inverted fluorescence microscope to confirm successful transduction.

### 2.7 RNA Sequencing

Briefly, 1.5 µg of total RNA per sample served as the input for library preparation. Library construction and subsequent sequencing were carried out following previously established protocols [29, 30]. Differential expression analysis was then performed using the DESeq2 R package (version 1.36.0), with differentially expressed genes (DEGs) defined by a stringent threshold of *p* < 0.01. RNA sequencing was performed on ANGPTL4‐overexpressing GC cell lines, and the raw sequencing data have been deposited in the GEO database under accession number GSE336544. The raw sequencing data in AGS cells with or without cANGPTL4 treatment have been deposited in the GEO database under accession number GSE336542. Raw RNA‐seq data of wild‐type HGC‐27 cells treated with conditioned medium from ANGPTL4‐overexpressing HGC‐27 cells and matched negative control cells have been deposited in the GEO database under accession number GSE338826.

### 2.8 RNA Isolation and Quantitative RT‐PCR

The qPCR analysis was conducted using One Step TB Green PrimeScriptTM RT‐PCR Kit II (cat No. RR086A, Takara, China). Each gene was run in triplicate. Relative fold changes in gene expression were calculated using the comparative ^ΔΔ^Ct method. The primer sequences in this study are as follows (indicated as 5′→3′): ANGPTL4_qF: TCTCTGGAGGCTGGTGGTTT; ANGPTL4_qR: AGAGTCACCGTCTTTCGTGG; CXCL8_qF: CCAGGAAGAAACCACCGGAA; CXCL8_qR: CTCCTTGGCAAAACTGCACC; GAPDH‐qF: TCACCAGGGCTGCTTTTA; GAPDH‐qR: AAGGTCATCCCTGAGCTGAA.

### 2.9 Western Blot Assay

The immunoblotting assays were performed as we described previously [27]. Briefly, the GC cell lines were lysed in RIPA buffer (Beyotime Biotechnology, Shanghai, China) with 1 mM PMSF (Beyotime Biotechnology, Shanghai, China). The protein concentration of all samples was quantified and normalized using BSA method. Then, the protein samples were electrophoresed and transferred to the PVDF membranes (Millipore, USA). After incubating with primary antibody and secondary antibody, the final images were visualized using a Imaging System (SCG‐W5000, ServiceBio, Wuhan, China). The primary antibodies used in this study were as follows: DYKDDDDK tag (20543‐1‐AP, Proteintech, Wuhan, China), GAPDH (10494‐1‐AP, Proteintech, Wuhan, China), ANGPTL4 (A2011, ABclonal, Wuhan, China), E‐cadherin (20874‐1‐AP, Proteintech, Wuhan, China), EPCAM (A23654, ABclonal, Wuhan, China), CLDN1 (A2196SP, ABclonal, Wuhan, China), Vimentin (10366‐1‐AP, Proteintech, Wuhan, China), SNAI1 (A5243, ABclonal, Wuhan, China), Twist1 (A15596, ABclonal, Wuhan, China), PARP1(13371‐1‐AP, Proteintech, Wuhan, China), Caspase‐3 (19677‐1‐AP, Proteintech, Wuhan, China), Annexin V(11060‐1‐AP, Proteintech, Wuhan, China), CXCL8 (27095‐1‐AP, Proteintech, Wuhan, China), RELB, (25027‐1‐AP, Proteintech, Wuhan, China), NFKB1 (14220‐1‐AP, Proteintech, Wuhan, China), NFKB2 (10409‐2‐AP, Proteintech, Wuhan, China), RELA (10745‐1‐AP, Proteintech, Wuhan, China).

### 2.10 Co‐Immunoprecipitation (Co‐IP) for Cell Lysate

AGS and HGC‐27 cells were transduced with ANGPTL4‐overexpressing lentivirus (GeneChem, Shanghai, China). After 48 h, cells (approximately 1 × 10^7^) were harvested and lysed in 1 mL of lysis buffer on ice for 60 min. Lysates were centrifuged at 12 000 rpm for 15 min at 4°C. A portion of the supernatant was retained as the input group and stored at −80°C overnight. The remaining supernatant was incubated with magnetic beads conjugated with mouse anti‐DDDDK tag monoclonal antibody (AE037, ABclonal, Wuhan, China) overnight at 4°C on a vertical rotator. After incubation, the beads were washed three times with wash buffer using a magnetic rack. SDS loading buffer was added to both the input lysate and the IP beads, followed by boiling at 100°C for 10 min. Samples were subsequently analyzed by western blotting.

### 2.11 Co‐Immunoprecipitation (Co‐IP) for Culture Supernatants

AGS and HGC‐27 cells were transduced with ANGPTL4‐overexpressing lentivirus and cultured for 48 h. The conditioned medium was collected, and a portion was retained as the supernatant input group. The remaining medium was incubated with magnetic beads conjugated with mouse anti‐DDDDK tag monoclonal antibody (AE037, ABclonal, Wuhan, China) overnight at 4°C on a vertical rotator. The beads were collected as the supernatant IP group. SDS loading buffer was added to both the input medium and the IP beads, followed by boiling at 100°C for 10 min. Samples were subsequently analyzed by western blotting. Immunoprecipitated complexes were subsequently resolved by SDS‐PAGE and visualized by western blotting. A 30 µL aliquot of the verified sample was subjected to mass spectrometry analysis at Guangzhou Saiqing Biotechnology Co., Ltd. (Guangzhou, China). Candidate ANGPTL4‐interacting proteins were identified by Venn diagram analysis comparing the mass spectrometry results of the negative control and ANGPTL4‐overexpressing groups. Proteins detected exclusively in the ANGPTL4‐overexpressing group were considered candidate ANGPTL4 interaction partners.

### 2.12 Neutrophil Chemotaxis Assay

The HL‐60 cells used in this study were purchased from Ubigene Biosciences (Guangzhou, China). Chemotaxis of differentiated HL‐60 (dHL‐60) cells was assessed using Transwell inserts with a 3‐µm pore size (Corning, Cat. No. 3415). Briefly, HL‐60 cells were treated with 1.25% DMSO in medium supplemented with 20% fetal bovine serum (FBS) for 5 days to induce neutrophil‐like differentiation. Before the assay, cells were washed with PBS prewarmed to 37°C and centrifuged at 1000 rpm for 3 min. Cells (1 × 10^5^ per insert) were resuspended in 200 µL of serum‐free RPMI‐1640 medium and added to the upper chamber. Control (NC) and ANGPTL4‐overexpressing (OE) HGC‐27 cells were cultured in T25 flasks until reaching approximately 50% confluence. The culture medium was then replaced with RPMI‐1640 medium containing 1% FBS, and cells were cultured for 48 h. Conditioned medium (CM) was collected and centrifuged at 2000 rpm for 5 min to remove cells. A total of 600 µL of CM was added to each lower chamber. For CXCL8 neutralization experiments, OE CM was preincubated with an anti‐human CXCL8 neutralizing antibody (0.5 µg mL^−1^; R&D Systems, Cat. No. AF‐208‐SP). Preincubation was performed at 37°C for 30 min before CM was added to the lower chamber. The plates were incubated for 3.5 h at 37°C in a humidified atmosphere containing 5% CO_2_. After incubation, the inserts were removed, and cells that had migrated into the lower chamber were collected. The collected cells were centrifuged at 1000 rpm for 5 min at room temperature. Cells were resuspended to a final volume of 100 µL and counted without dilution using a hemocytometer. All nine large squares of the counting chamber were counted. The total number of migrated cells was calculated using the following formula: Migrated cells = N/9*10000*0.1. N represents the total cell count across the nine large squares, and 0.1 represents the final resuspension volume in milliliters. The assay was performed in three independent experiments, with all three groups tested using the same dHL‐60 cell batch within each experiment.

### 2.13 Mouse Xenograft Model

The study protocol was approved by the Laboratory Animal Ethics Committee of Hubei University of Medicine. Four‐week‐old female BALB/c nude mice (RRID: IMSR_JAX:002019) were purchased from the Laboratory Animal Center of Hubei University of Medicine (Shiyan, Hubei, China). ANGPTL4‐overexpressing HGC‐27 cells and corresponding negative control cells were cultured, harvested, and resuspended in a mixture of PBS and SWE Matrigel (Cat. No. G4130‐5ML, Servicebio, Wuhan, China). The nude mice were randomly divided into three groups: the control group received subcutaneous injections of negative control cells, while both the experimental group and the rescue group received equivalent subcutaneous injections of ANGPTL4‐overexpressing cells. Starting on day 9 post‐inoculation, the rescue group was administered subcutaneous injections of DNase I (2.5 mg kg^−1^, Cat. 10104159001, Merck, USA) daily for 12 consecutive days. Tumor volume and body weight were measured every 3 days starting from day 6 post‐inoculation. On day 21 post‐inoculation, all mice were euthanized, and subcutaneous tumors were excised for weighing, measurement, and subsequent analyses.

To evaluate the in vivo efficacy of recombinant human cANGPTL4, wild‐type AGS cells were subcutaneously inoculated into nude mice. Beginning on day 6 post‐inoculation, tumor volumes and body weights were monitored every 3 days. On day 9, mice were randomized into two groups. The treatment group was administered recombinant human cANGPTL4 (5 mg kg^−1^) every other day for 10 injections, whereas the control group received the vehicle. 18 days post‐inoculation, all mice were euthanized, and the excised subcutaneous tumors were weighed and measured for further downstream analyses. The tumor volume was calculated using the following formula: volume  =  length × (width)^2^/2.

### 2.14 Multiplex IHC Assay

Multiplex IHC assay was conducted using mIF Kits (Servicebio Technology, Wuhan, China) according to the manufacturer's protocol. Multicolor immunofluorescence staining leverages tyramide signal amplification (TSA) technology to simultaneously detect multiple distinct proteins on a single tissue section. The primary antibodies and corresponding TSA types used in this study were as follows: E‐cad (GB12083, Servicebio, Wuhan, China), VIM (GB121308, Servicebio, Wuhan, China), Ly6G (GB11229, Servicebio, Wuhan, China). For the IHC assay in GC tissues, tissue sections were stained with primary antibodies against MPO (GB120016, Servicebio, Wuhan, China) and ANGPTL4 (A2011, ABclonal, Wuhan, China) overnight at 4°C, followed by standard secondary antibody incubation and DAB visualization.

### 2.15 Statistical Analysis

For gene expression analysis of different subtypes of GC, the *p* values were estimated using Mann–Whitney nonparametric test. Survival curves were calculated using the Kaplan–Meier method, and differences between the curves were analyzed using the log‐rank test. All the rest of the experiments used unpaired *t*‐test or a one‐way ANOVA test. All tests with p values less than 0.05 are considered statistically significant.

## 3 Results

### 3.1 Identification of Genes with DNA Hypermethylation and Low Expression in EBVaGC

Based on the multi‐omics molecular classification analysis of the TCGA database, GC can be further subclassified into five subtypes: Epstein‐Barr virus‐positive (EBV), microsatellite instability (MSI), chromosomal instability (CIN), and genomically stable (GS), and DNA polymerase ε mutation (POLE) [14]. Using the LinkedOmics online platform, we systematically evaluated the differences in gene expression and DNA methylation across different molecular subtypes of GC. Notably, in the EBVaGC subtype, the genes with the most significantly upregulated DNA methylation and the most significantly downregulated expression levels are presented in expression and DNA methylation heatmaps, respectively (Figure 1A,B). Given that DNA methylation typically negatively regulates gene expression, we further identified genes with significantly upregulated DNA methylation and significantly downregulated expression levels in EBVaGC using a Venn diagram (Figure 1C). For instance, the expression level of ANGPTL4 is significantly downregulated, while its DNA methylation level is markedly upregulated in EBVaGC (Figure 1D,E).

**FIGURE 1:**
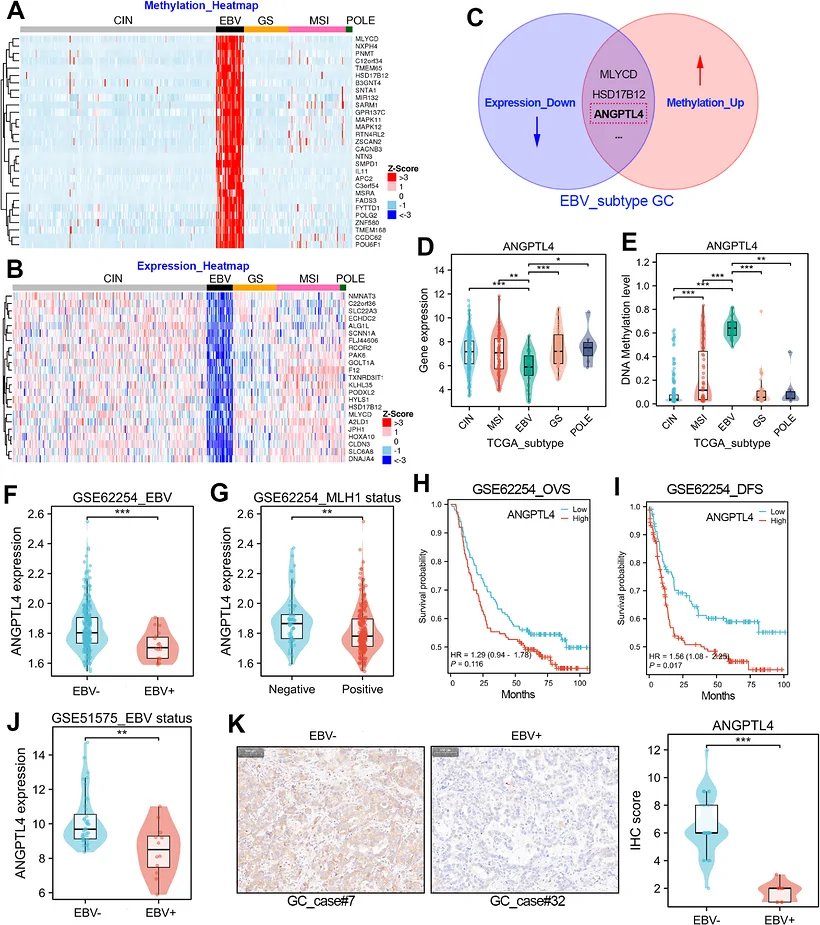
ANGPTL4 is downregulated in EBVaGC. (A) DNA methylation analysis was performed across different gastric cancer subtypes in the TCGA‐STAD cohort. The heatmap displays genes with the most significantly upregulated DNA methylation in the EBV subtype. (B) Differential gene expression analysis was performed across different gastric cancer subtypes in the TCGA‐STAD cohort. The heatmap displays the genes with the most significantly downregulated expression in the EBV subtype. (C–E) *ANGPTL4* is one of the most significantly downregulated genes with the highest degree of DNA hypermethylation in EBVaGC. (F,G) In the GSE62254 cohort, ANGPTL4 expression was significantly downregulated in EBV‐positive or MLH1‐mutation‐positive GC tissues. (H,I) High ANGPTL4 expression was clinically associated with a poor prognosis in the GSE62254 cohort. (J) *ANGPTL4* was significantly downregulated in EBV‐positive gastric cancer tissues in the GSE51575 cohort. (K) IHC staining of our in‐house GC cohort demonstrated a significantly lower ANGPTL4 protein level in EBVaGC compared with EBVnGC tissues. *p < 0.05; **p < 0.01; ***p < 0.001.

In the GSE62254 cohort, we similarly observed that ANGPTL4 is significantly downregulated in EBVaGC (Figure 1F). Furthermore, ANGPTL4 expression is markedly reduced in GC patients harboring MLH1 mutations (Figure 1G). Despite this, in EBVnGC, patients with high ANGPTL4 expression exhibit a poorer prognosis (Figure 1H,I). Consistently, ANGPTL4 expression is significantly downregulated in EBVnGC patients in the GSE51575 cohort (Figure 1J). In addition, we validated the aforementioned findings in our in‐house GC cohort using IHC assay, demonstrating that ANGPTL4 expression is significantly lower in EBV‐positive GC than in EBV‐negative GC (Figure 1K).

### 3.2 EBV Infection Silences ANGPTL4 Expression via DNA Hypermethylation in EBVaGC

According to UCSC web tool, a CpG island containing nine CpG sites is located in the promoter region of ANGPTL4 (Figure 2A). Correlation analysis revealed that the DNA methylation levels of all nine CpG sites are significantly negatively correlated with ANGPTL4 expression in GC (Figure 2A,B). Consistent with our expectations, the methylation levels of all nine CpG sites in the ANGPTL4 promoter region are markedly upregulated in EBV‐positive GC tissues from the GSE99111 cohort (Figure 2C). Furthermore, ANGPTL4 expression is markedly lower in EBV‐positive cell lines compared with EBV‐negative cell lines (Figure 2D). Subsequently, we analyzed the temporal dynamics of ANGPTL4 promoter DNA methylation in the normal gastric epithelial cell line GES‐1 following EBV infection using the GSE89269 dataset. Notably, the methylation levels of all nine CpG sites in the ANGPTL4 promoter region surge dramatically approximately two weeks post‐EBV infection (Figure 2E). Correspondingly, RNA‐seq analysis revealed that ANGPTL4 expression levels decrease progressively in GES‐1 cells as the duration of EBV infection increases (Figure 2F). In EBVnGC cell lines HGC‐27 and AGS, we similarly observed a significant downregulation of ANGPTL4 expression following EBV infection (Figure 2G–I). Collectively, these results indicate that EBV infection downregulates ANGPTL4 expression via DNA hypermethylation.

**FIGURE 2:**
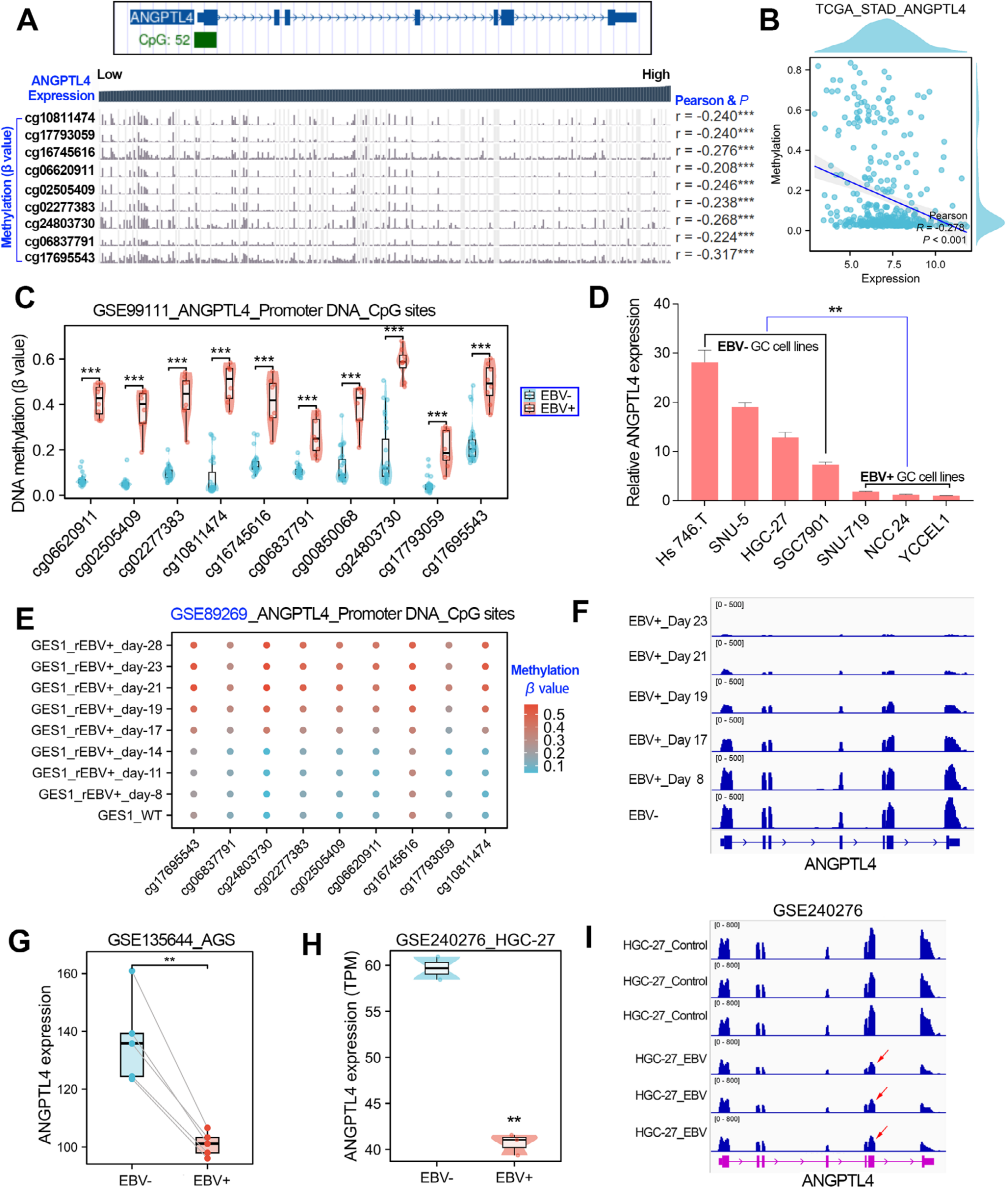
EBV infection silences ANGPTL4 expression in GC cells via DNA hypermethylation. (A, B) The DNA methylation levels of all CpG sites in the *ANGPTL4* promoter region were significantly negatively correlated with its expression levels in the TCGA‐STAD cohort. (C) DNA methylation at the ANGPTL4 promoter was significantly elevated in EBVaGC compared with EBVnGC. (D) *ANGPTL4* expression levels were significantly lower in EBV‐positive gastric cancer cell lines than in EBV‐negative cell lines. (E) An EBV infection model was established using the normal gastric epithelial cell line GES‐1. The dynamic changes in DNA methylation levels at the ANGPTL4 promoter were evaluated at various time points post‐infection. (F) Changes in ANGPTL4 expression levels in GES‐1 cells at different time points post‐EBV infection. (G‐I) EBV infection models were established in AGS and HGC‐27 cells, respectively. Transcriptomic sequencing results demonstrated that EBV infection significantly downregulated ANGPTL4 expression. **p < 0.01; ***p < 0.001.

### 3.3 ANGPTL4 Is Overexpressed and Predicted Poor Prognosis in EBVnGC

Given that EBVaGC accounts for less than 10% of all gastric cancers, this study focuses on elucidating the biological functions of ANGPTL4 in EBVnGC. Using IHC, we assessed ANGPTL4 protein expression in tumor versus adjacent non‐tumor tissues within the EBVnGC cohort (Figure 3A). The results showed that ANGPTL4 protein expression was significantly upregulated in EBVnGC (Figure 3B). Besides, we found that ANGPTL4 mRNA level was significantly upregulated in the GSE122401 and GSE184336 cohorts (Figure 3C,D).

**FIGURE 3:**
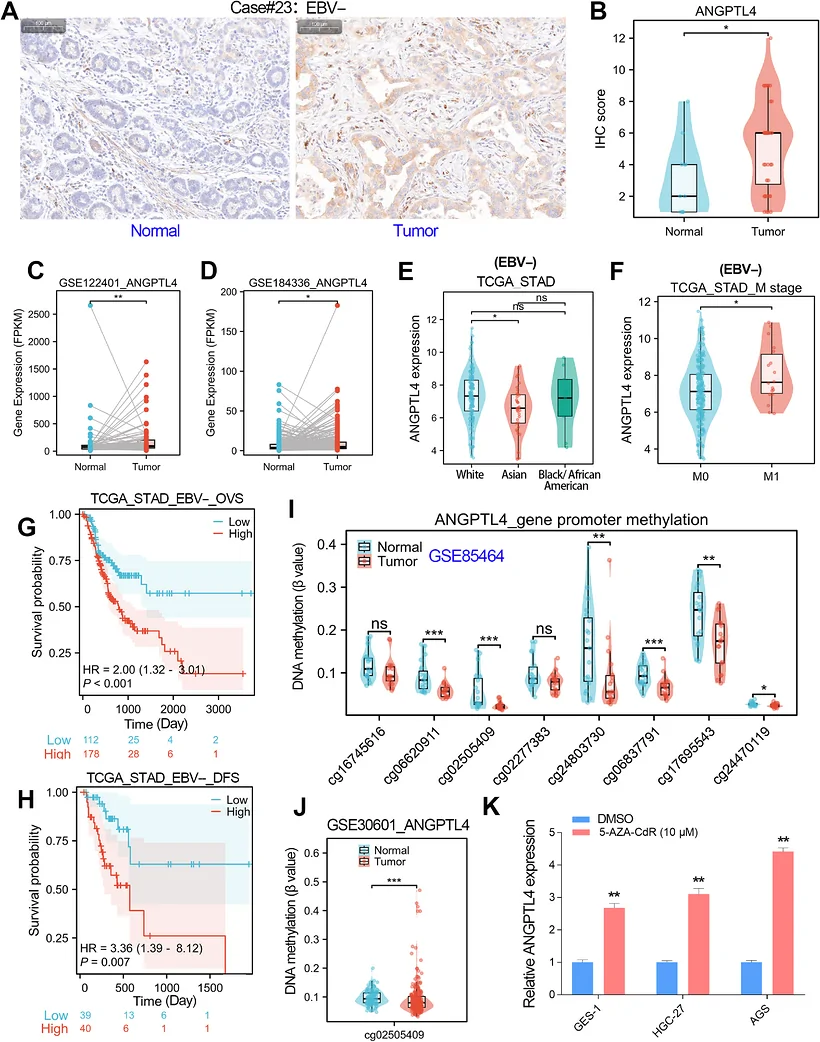
*ANGPTL4* was significantly upregulated in EBVnGC and correlated with poor clinical outcomes. (A,B) IHC staining demonstrated a significant upregulation of ANGPTL4 protein in EBVnGC tumor tissues. (C,D) In the GSE122401 (*n* = 80) and GSE184336 (*n* = 230) gastric cancer cohorts, ANGPTL4 transcript levels were significantly upregulated in tumor tissues. (E) After excluding the EBV subtype in the TCGA‐STAD cohort, ANGPTL4 expression was significantly associated with ethnicity in GC patients. (F) In the TCGA cohort, ANGPTL4 expression was significantly associated with distant metastasis in EBVnGC patients. (G,H) In the TCGA cohort, EBVnGC patients with high *ANGPTL4* expression exhibited significantly shorter overall survival (OS) and disease‐free survival (DFS). (I,J) In the GSE85464 and GSE30601 cohorts, DNA methylation levels at the *ANGPTL4* promoter were significantly decreased in tumor tissues compared with normal tissues. (K) ANGPTL4 expression was significantly induced by DNA methyltransferase inhibitor treatment in GES‐1 and GC cell lines. *p < 0.05; **p < 0.01; ***p < 0.001.

After excluding EBV subtype samples from the TCGA cohort, the remaining four subtypes in the TCGA cohort were merged to define the EBVnGC cohort, and the clinical significance of ANGPTL4 was subsequently analyzed. We found that ANGPTL4 expression is higher in Caucasian patients and is significantly positively correlated with distant metastasis (Figure 3E,F). Consistently, EBVnGC patients with high ANGPTL4 expression exhibited poorer overall (OVS) and disease‐free survival (DFS, Figure 3G,H).

Given that ANGPTL4 transcripts are also significantly upregulated in GC, we further analyzed the differential DNA methylation levels of CpG sites in the ANGPTL4 promoter between normal and tumor tissues. Strikingly, in the GSE85464 and GSE30601 cohort, the methylation levels of the ANGPTL4 promoter DNA were significantly lower in GC tissues than in normal tissues (Figure 3I,J). Treatment with a DNA methyltransferase inhibitor (5‐Aza‐CdR) similarly resulted in a significant activation of ANGPTL4 expression in normal gastric epithelial cell line GES‐1 and different GC cell lines (Figure 3K). These results suggest that promoter hypomethylation significantly upregulates ANGPTL4 expression in EBVnGC, which is clinically correlated with poor prognosis and distant metastasis.

### 3.4 ANGPTL4 Overexpression Promotes GC Progression in Vitro

To investigate the biological function of ANGPTL4 in GC, we conducted gain‐of‐function studies in HGC‐27 and AGS cell lines and loss‐of‐function studies in HGC‐27 cell line. qRT‐PCR and western blot analysis confirmed the successful establishment of ANGPTL4‐overexpressing cell lines (Figure 4A,B). The CCK‐8 assays showed that ANGPTL4 overexpression significantly accelerated tumor cell growth in GC cell lines (Figure 4C,D). Similarly, EdU staining and colony formation assays indicated that ANGPTL4 overexpression enhanced the proliferation of GC cell lines (Figure 4E,F). Conversely, ANGPTL4 overexpression significantly inhibited apoptosis, as evidenced by the TUNEL assay and the decreased levels of cleaved PARP1 and CASPASE3 in GC cell lines (Figure 4G,H). Moreover, wound healing and transwell invasion assays revealed that ANGPTL4 overexpression significantly promoted the migration and invasion of GC cell lines (Figure 4I,J). Conversely, knockdown of ANGPTL4 significantly inhibited the proliferation and invasion of HGC‐27 cells (Figure 4K–P).

**FIGURE 4:**
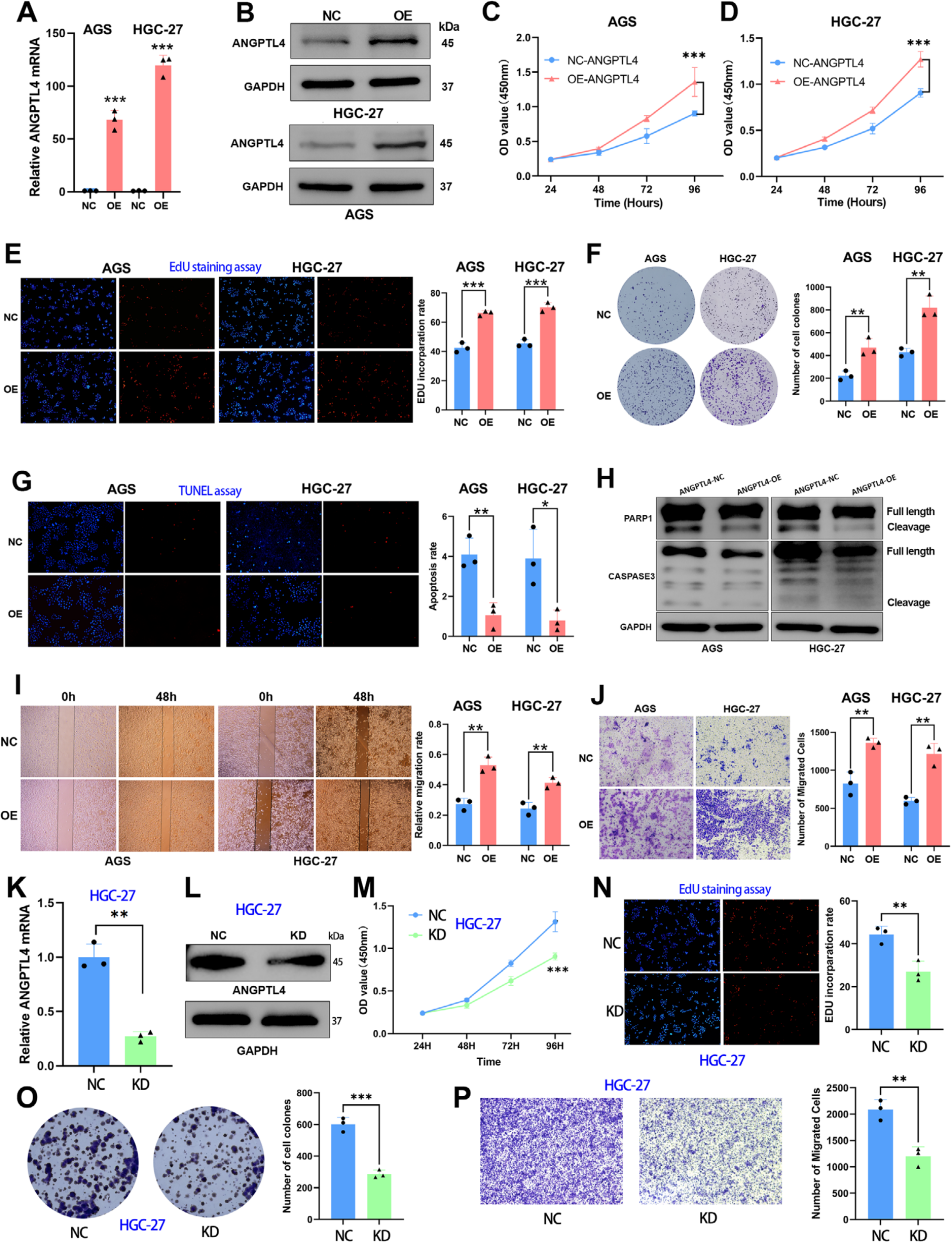
ANGPTL4 plays an oncogenic role in GC. (A,B) Overexpression efficiency of ANGPTL4 in gastric cancer stable cell lines was verified by qRT‐PCR and western blotting assays. (C,D) The CCK‐8 assay was performed to evaluate the effect of *ANGPTL4* overexpression on the growth rate of AGS and HGC‐27 cells. (E) The EdU staining assay was performed to evaluate the effect of ANGPTL4 overexpression on the proliferation of AGS and HGC‐27 cells. (F) Colony formation assays demonstrated that *ANGPTL4* overexpression significantly enhanced the proliferation capacity of GC cell lines. (G,H) Overexpression of *ANGPTL4* markedly suppressed apoptosis in GC cell lines. (I,J) Wound healing and Transwell assays demonstrated that *ANGPTL4* overexpression significantly enhanced the migration and invasion capacities of GC cell lines. (K,L) Knockdown efficiency of ANGPTL4 in HGC‐27 cells was verified by qRT‐PCR and western blotting assays. (N‐P) ANGPTL4 knockdown significantly inhibited HGC‐27 cell proliferation and invasion. *p < 0.05; **p < 0.01; ***p< 0.001.

### 3.5 ANGPTL4 Overexpression Activates EMT Signaling in GC

According to our previous findings, ANGPTL4 expression was positively correlated with distant metastasis in GC, and its overexpression significantly promoted GC cell migration and invasion. The EMT signaling pathway is closely associated with cancer metastasis [31]. Thus, to initiate our investigation into the correlation between ANGPTL4 and the EMT signaling pathway, we first categorized GC cell lines into two distinct subtypes based on the expression of EMT‐related genes: mesenchymal‐like cells (characterized by high expression of mesenchymal markers) and epithelial‐like cells (characterized by high expression of epithelial markers, Figure 5A). ANGPTL4 expression was markedly higher in mesenchymal‐like cells than in epithelial‐like cells (Figure 5B,C). Furthermore, in the TCGA‐STAD cohort, ANGPTL4 expression was highly positively correlated with mesenchymal marker genes (Figure 5D). Western blot analysis further demonstrated that ANGPTL4 overexpression significantly activated the EMT signaling pathway in GC cell lines (Figure 5E,F). In vivo validation using a nude mouse xenograft model confirmed that ANGPTL4 overexpression significantly promoted tumor growth (Figure 5G). Consistently, ANGPTL4 overexpression significantly suppressed the expression of the epithelial marker E‐cadherin while promoting the expression of the mesenchymal marker Vimentin in the xenograft tumors (Figure 5H,I). Collectively, these findings demonstrate that ANGPTL4 positively regulates the EMT signaling pathway in GC.

**FIGURE 5:**
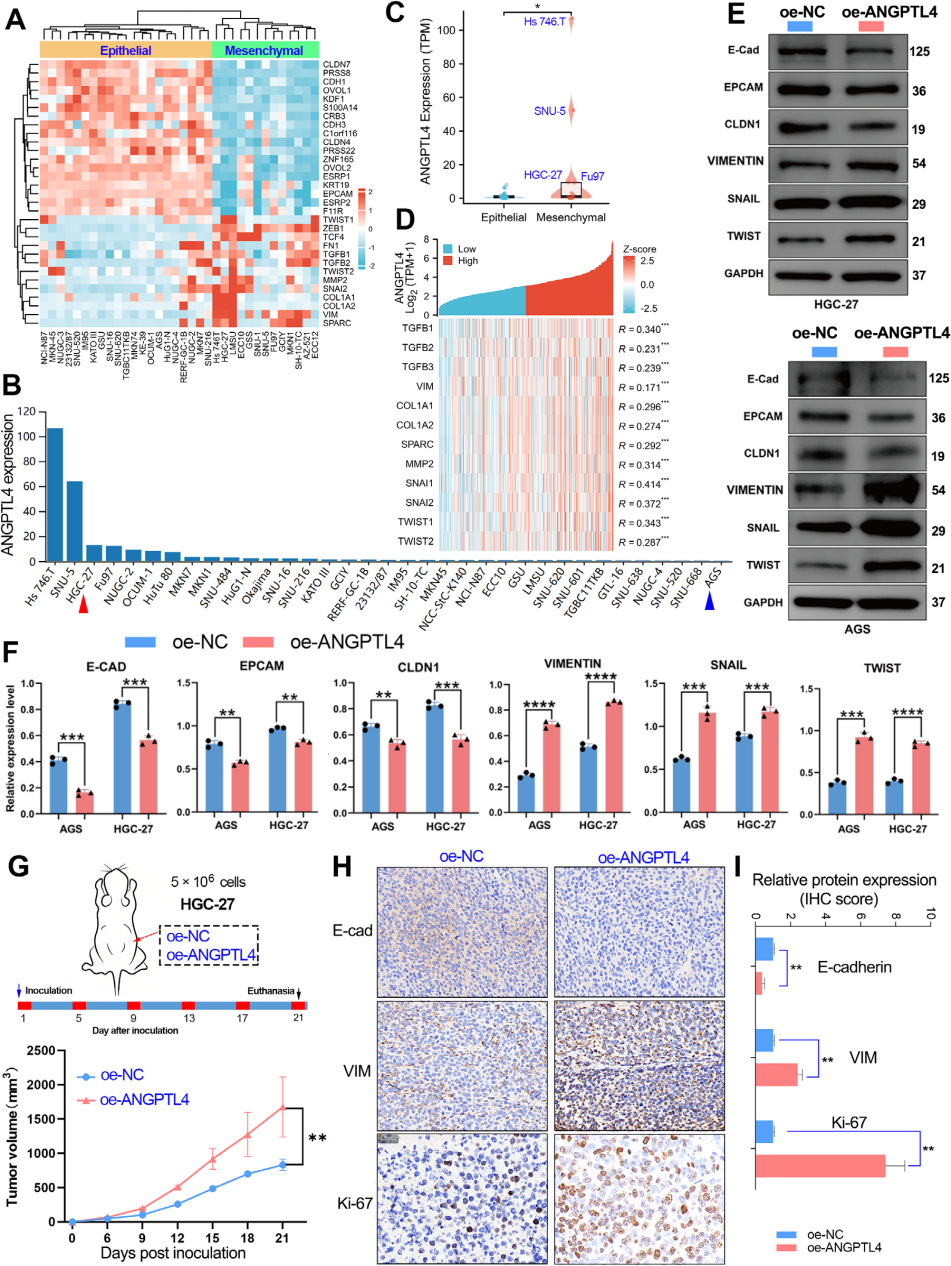
In vitro and in vivo assays confirmed that *ANGPTL4* overexpression significantly activates EMT signaling in GC cells in vitro and in vivo. (A) Based on the expression patterns of EMT‐related marker genes, we classified different GC cell lines into two major categories: mesenchymal‐like subtype and epithelial‐like subtype. (B) Bar charts showing the expression levels of ANGPTL4 in different gastric cancer cells based on the HPA dataset. (C) *ANGPTL4* exhibited significantly higher expression levels in mesenchymal‐like gastric cancer cell lines compared to epithelial‐like cell lines. (D) In the TCGA‐STAD cohort, the expression level of ANGPTL4 was significantly co‐expressed with mesenchymal marker genes. (E,F) Overexpression of *ANGPTL4* markedly triggered the EMT signaling cascade in GC cell lines. (G) *ANGPTL4* overexpression significantly enhanced the growth rate of xenograft tumors in nude mice. The volume of xenograft tumors in the ANGPTL4 overexpression group was significantly larger than that in the control group. (H,I) IHC staining of xenograft tumors revealed that *ANGPTL4* overexpression significantly downregulated the expression of the epithelial marker E‐cadherin, while markedly upregulating the mesenchymal marker Vimentin and the proliferation marker Ki‐67. *p < 0.05; **p < 0.01; ***p < 0.001.

### 3.6 ANGPTL4 Is Involved in Neutrophil Infiltration in GC

The GSE122401 cohort includes bulk RNA‐seq data from 80 paired tumor and adjacent normal tissue samples. Using single‐sample gene set enrichment analysis (ssGSEA), we generated a heatmap to illustrate the correlation between ANGPTL4 expression and the infiltration levels of various immune cell types in this cohort (Figure 6A). Notably, GC exhibits an immunosuppressive microenvironment, characterized by a significant decrease in CD8+ T cells, B cells, and CD56bright NK cells, along with a marked increase in the proportions of neutrophils, macrophages, and regulatory T cells (Tregs, Figure 6A). Obviously, ANGPTL4 expression was positively correlated with neutrophil infiltration, and both were significantly upregulated in GC tissues (Figure 6A–C). Consistently, in the TCGA‐STAD cohort, ANGPTL4 expression was also significantly positively correlated with neutrophil infiltration (Figure 6D). However, it remains unclear whether this positive correlation is direct or indirect. To address this, we performed spatial transcriptomic analysis (GSE251950, Figure S1) in GC. Strikingly, in GC sample (ID: GSM7990477), regions with high ANGPTL4 expression (indicated by blue arrows) exhibited a pronounced enrichment of neutrophils (labeled by S100A9, Figure 6E). Furthermore, we found that GC samples with high ANGPTL4 expression displayed a substantially higher distribution of neutrophils, as indicated by the MPO marker (Figure 6F,G). In addition, analysis revealed that neutrophils were enriched in the peritumoral stromal region (Figure S2), suggesting direct communication between neutrophils and cancer cells. These findings suggest that ANGPTL4 protein likely directly participates in the infiltration of neutrophils into GC.

**FIGURE 6:**
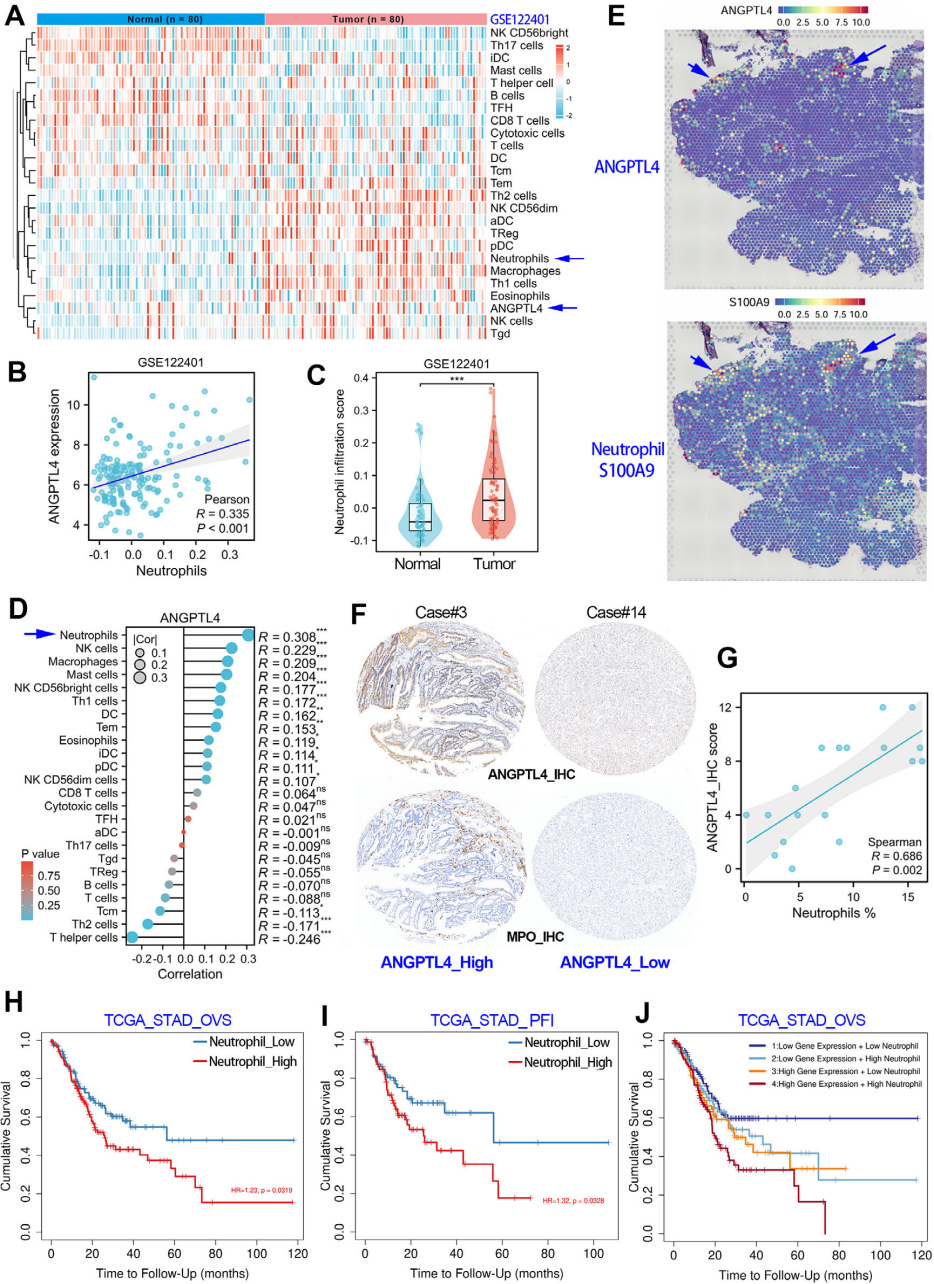
ANGPTL4 expression positively correlated with the degree of neutrophil infiltration, with both showing spatial co‐localization in GC tissues. (A) Immune infiltration analysis of the GSE122401 bulk transcriptome dataset was performed using the ssGSEA algorithm. The heatmap illustrates the distribution of *ANGPTL4* expression levels and the infiltration degrees of various immune cells in normal and tumor samples. (B) The expression level of *ANGPTL4* was significantly positively correlated with the degree of neutrophil infiltration. (C) Neutrophil infiltration was significantly increased in tumor tissues of the GSE122401 cohort. (D) In the TCGA‐STAD cohort, the expression level of ANGPTL4 was significantly positively correlated with the degree of neutrophil infiltration. (E) Spatial transcriptomic analysis revealed that neutrophils (identified by the marker S100A9) were significantly enriched in *ANGPTL4*‐highly expressed regions. (F,G) IHC staining assay was performed on serial tissue sections to verify the distribution patterns and correlation between ANGPTL4 expression and the neutrophil marker MPO in GC tissues. (H,I) Survival analysis via the TIMER3.0 web tool revealed that high levels of neutrophil infiltration in gastric cancer predict an unfavorable prognosis. (J) Survival analysis using the TIMER3.0 web tool indicated that gastric cancer patients with both high ANGPTL4 expression and high neutrophil infiltration had the worst prognosis. *p < 0.05; **p < 0.01.

Subsequent prognostic analysis revealed that GC tissues with high neutrophil infiltration were associated with significantly shorter overall survival (OS) and progression‐free survival (PFS, Figure 6H,I). Moreover, patients with both high ANGPTL4 expression and high neutrophil infiltration had the poorest prognosis, whereas those with low expression of both exhibited the best outcomes (Figure 6J). Collectively, these results preliminarily suggest that ANGPTL4 may drive GC progression by promoting neutrophil infiltration.

### 3.7 ANGPTL4 Overexpression Promotes Neutrophils Infiltration via Activation of NF‐κB/CXCL8 Axis in GC

To validate the role of ANGPTL4 in neutrophil infiltration in GC, we performed immunofluorescence staining in nude mouse xenograft tumors using an anti‐Ly6G antibody to label neutrophils (Figure 7A). As expected, ANGPTL4 overexpression significantly enhanced the infiltration of neutrophils within the xenograft tumors (Figure 7B). To elucidate the underlying molecular mechanisms by which ANGPTL4 promotes neutrophil infiltration, we conducted RNA sequencing analysis in HGC‐27 cells (Figure 7C). GO/KEGG analysis revealed that genes significantly upregulated following ANGPTL4 overexpression were predominantly enriched in the neutrophil migration and chemotaxis signaling pathways (Figure 7D). Notably, ANGPTL4 overexpression markedly promoted the mRNA expression of the chemokine CXCL8 in GC cell lines (Figure 7E). Given that the NF‐κB signaling pathway has been reported to be involved in the transcriptional regulation of CXCL8 [32], we performed correlation analysis and found that ANGPTL4/CXCL8 was highly co‐expressed with NF‐κB transcription factors in GC (Figure 7F). Furthermore, Western blot analysis demonstrated that ANGPTL4 overexpression significantly upregulated the protein levels of RELA, RELB, NFKB1/2, and CXCL8 in GC cell lines (Figure 7G). Transcriptomic data also showed that RELA overexpression significantly increased CXCL8 transcript levels in GC cell lines (Figure 7H,I). Therefore, ANGPTL4 primarily promotes the mRNA and protein expression of CXCL8 by activating the NF‐κB signaling pathway. In addition, neutrophil chemotaxia assay further confirmed that ANGPTL4 overexpression promotes neutrophil migration through CXCL8 secretion (Figure 7J). Taken together, ANXA5 overexpression promotes neutrophils recruitment via NF‐Κb/CXCL8 axis.

**FIGURE 7:**
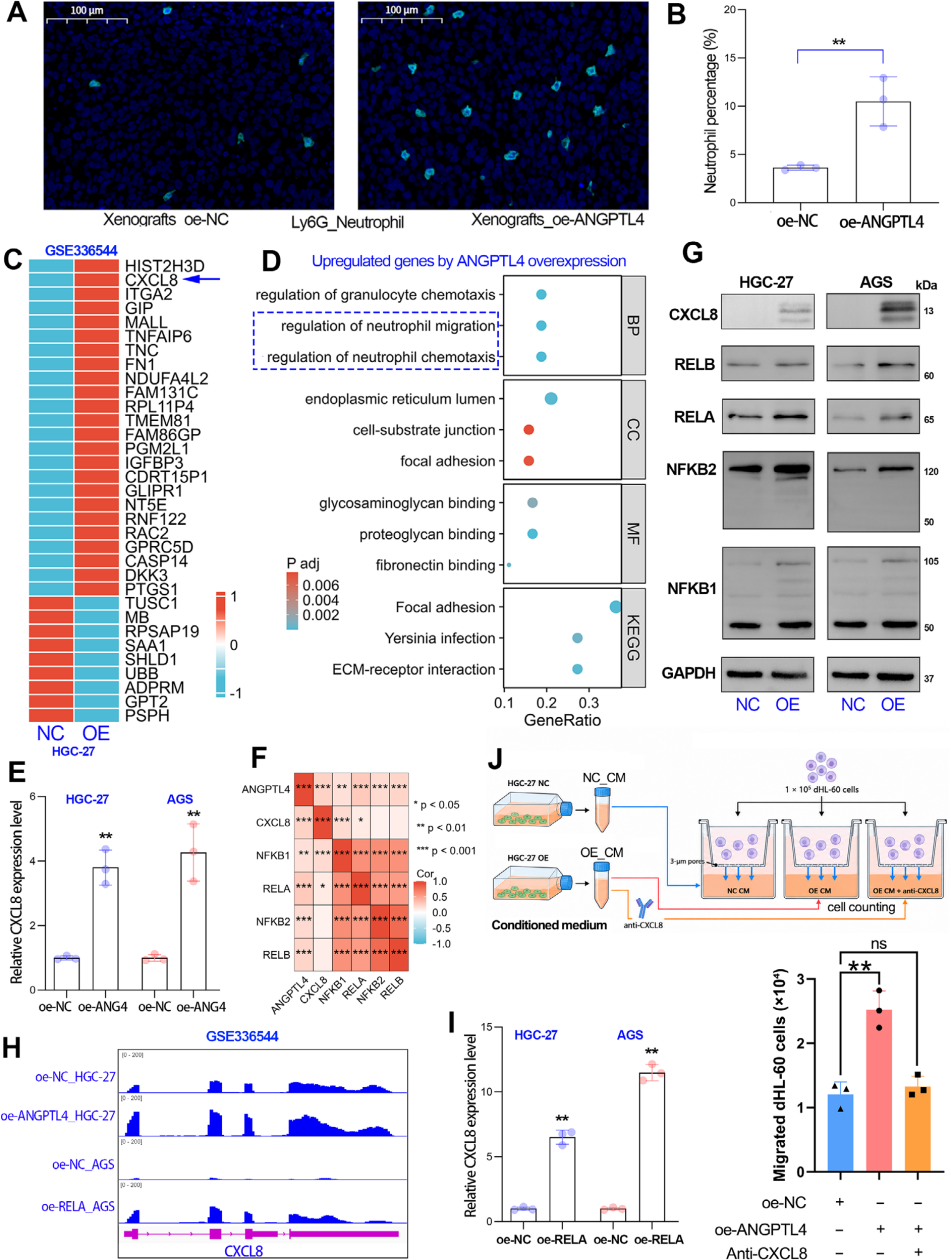
ANGPTL4 drives neutrophils recruitment by activating NF‐κB/CXCL8 axis in GC. (A,B) In the subcutaneous xenograft model in nude mice, the level of neutrophil infiltration in the transplanted tumors was significantly higher in the *ANGPTL4* overexpression group compared to the control group. (C) RNA sequencing was performed on ANGPTL4‐overexpressing HGC‐27 cells. The most significantly differentially expressed genes (DEGs) following ANGPTL4 overexpression are presented in the heatmap. (D) GO and KEGG enrichment analyses revealed that the significantly upregulated genes following *ANGPTL4* overexpression were mainly enriched in neutrophil chemotaxis and migration pathways. (E) Overexpression of *ANGPTL4* resulted in a significant increase in the mRNA expression of the chemokine *CXCL8*. (F) In the TCGA‐STAD cohort, the expression level of *ANGPTL4*/*CXCL8* was highly co‐expressed with transcription factors of the NF‐κB signaling pathway. (G) Western blot results confirmed that *ANGPTL4* overexpression significantly activated the NF‐κB signaling pathway and upregulated the protein expression level of *CXCL8*. (H, I) Ectopic expression of *RELA* resulted in a significant increase in *CXCL8* mRNA expression in GC cell lines. (J) Neutrophil chemotaxis assay verified that conditioned medium of the *ANGPTL4*‐overexpression HGC‐27 cells promoted neutrophil migration and can be rescued by CXCL8 neutralizing antibodies. *p < 0.05; **p < 0.01; ***p < 0.001.

### 3.8 Secreted cANGPTL4 is Sufficient to Activate the EMT Signaling Pathway and Promote Neutrophil Infiltration in GC

ANGPTL4 is a secreted protein that, upon extracellular release, can be cleaved by proprotein convertases into two active fragments: the C‐terminal (∼37 kDa, cANGPTL4) and the N‐terminal (nANGPTL4, Figure 8A). However, the predominant functional form of ANGPTL4 in GC remains unclear. Therefore, we introduced a Flag tag to the C‐terminus of ANGPTL4 and performed co‐immunoprecipitation (Co‐IP) and Western blot analyses on both whole‐cell lysates and culture supernatants (Figure 8B). In both mesenchymal‐like HGC‐27 and epithelial‐like AGS cells, intracellular ANGPTL4 existed primarily as a full‐length protein, whereas the culture supernatant contained predominantly cANGPTL4 along with a minor fraction of full‐length ANGPTL4 (Figure 8C–F). In addition, transcriptomic analysis of wild‐type HGC‐27 cells treated with conditioned media (CM) from ANGPTL4‐overexpressing or control HGC‐27 cells showed that ANGPTL4‐derived CM significantly upregulated the expression of EMT‐related transcription factors (ZEB1/2, SNAI1/2, and TWIST1/2) in recipient cells (Figure S3A–C). These results suggest that the activation of downstream EMT signaling pathways by ANGPTL4 is dependent on its secretion.

**FIGURE 8:**
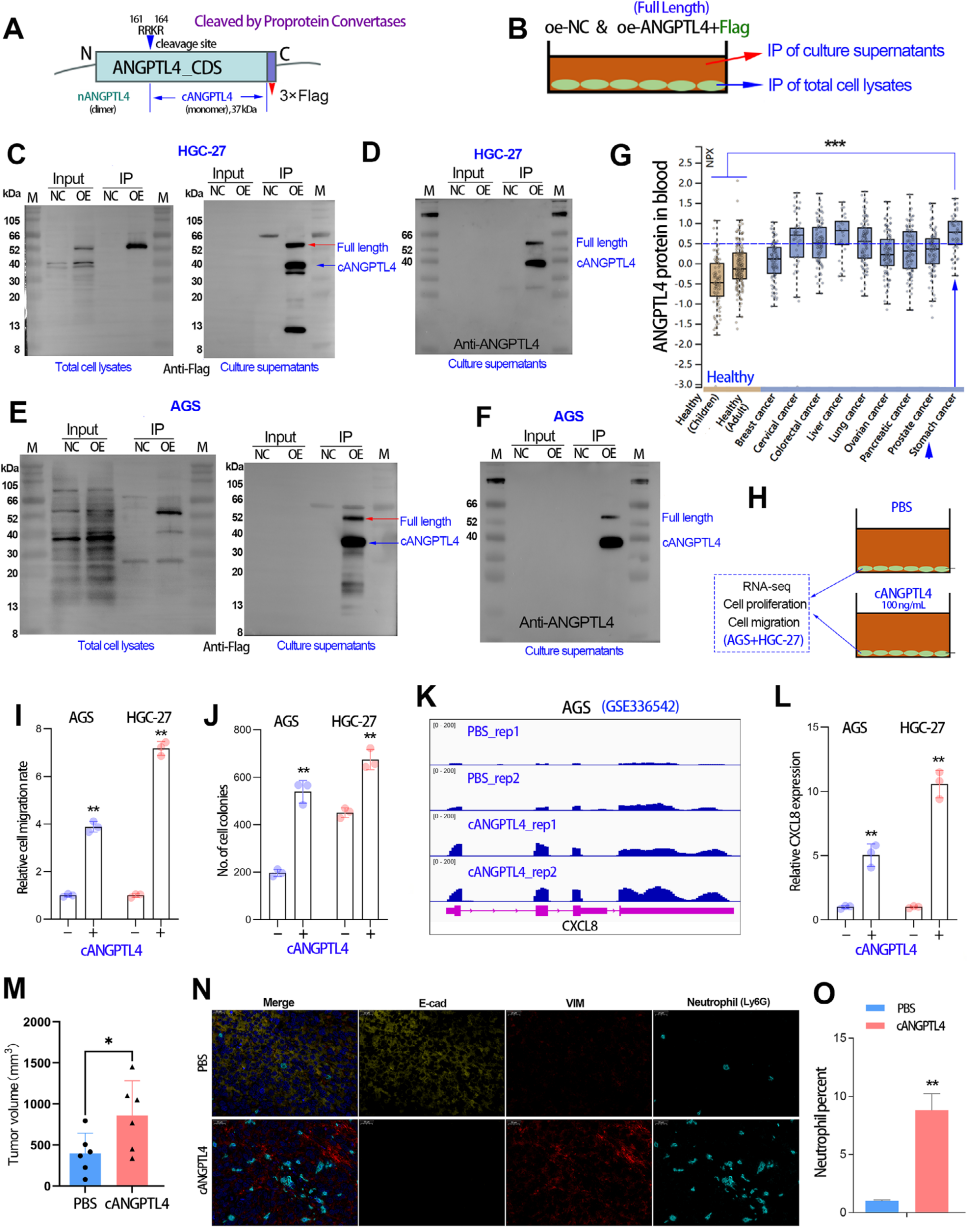
*cANGPTL4* treatment is sufficient to facilitate the proliferation, migration, and EMT of GC cells. (A,B) *ANGPTL4* is a secreted protein that can be cleaved by proprotein convertases into N‐terminal and C‐terminal fragments. To evaluate the secretion characteristics of *ANGPTL4* in GC, we performed transfection experiments using full‐length *ANGPTL4* tagged with a 3×FLAG at the C‐terminus. Co‐immunoprecipitation (Co‐IP) assays were then conducted in both cell lysates and culture supernatants using an anti‐FLAG antibody. (C–F) In both mesenchymal HGC‐27 and epithelial AGS gastric cancer cell lines, ANGPTL4 exists primarily as a full‐length protein intracellularly. Extracellularly, ANGPTL4 is present as both the full‐length form and the C‐terminal fragment (cANGPTL4), with cANGPTL4 being the predominant secreted form. (G) Based on the HPA datasets, Serum *ANGPTL4* protein expression was significantly increased in GC patients. (H) Schematic diagram illustrating the treatment of GC cells with recombinant human cANGPTL4 protein. (I,J) Treatment with cANGPTL4 significantly enhanced the proliferation and migration capabilities of GC cell lines. (K,L) RNA sequencing and qRT‐PCR assays revealed that cANGPTL4 treatment significantly upregulated the expression level of the chemokine *CXCL8* in GC cell lines. (M) Subcutaneous xenograft assays in nude mice confirmed that cANGPTL4 treatment significantly enhanced tumor proliferation. (N,O) Multiplex immunohistochemistry (mIHC) results confirmed that cANGPTL4 treatment significantly activated the EMT signaling pathway and enhanced neutrophil infiltration in xenograft tumors. *p < 0.05; **p < 0.01; ***p < 0.001.

Data from the Human Protein Atlas (HPA) revealed that circulating ANGPTL4 levels were significantly elevated in GC patients (Figure 8G). Given that cANGPTL4 is the major secreted form, we treated GC cell lines with recombinant human cANGPTL4 to evaluate its effects on cell behavior, the EMT signaling pathway, and neutrophil infiltration. As expected, cANGPTL4 treatment significantly promoted the migration and proliferation of GC cells (Figure 8H–J). Furthermore, RNA‐seq analysis and qRT‐PCR assays demonstrated that cANGPTL4 treatment was sufficient to upregulate CXCL8 expression in the HGC‐27 and AGS cell lines (Figure 8K,L). In vivo xenograft assays further confirmed that cANGPTL4 treatment markedly accelerated tumor growth in vivo (Figure 8M and Figure S4A–D). Multiplex immunohistochemistry (mIHC) assays revealed that cANGPTL4 treatment significantly activated the EMT signaling pathway and enhanced neutrophil infiltration within the xenograft tumors (Figure 8N,O). Collectively, these findings demonstrate that ANGPTL4 functions primarily in its secreted form cANGPTL4. Specifically, cANGPTL4 drives GC progression by activating the EMT signaling pathway and promoting neutrophil infiltration.

### 3.9 cANGPTL4 Interacts with ANXA5 to Enhance Its Protein Stability, Thereby Promoting NETosis and NET Release in GC

IHC staining revealed that MPO‐positive staining areas of tumor‐infiltrating neutrophils extended far beyond the neutrophil cell body and presented a diffuse reticular pattern, strongly indicating the formation of NETs (Figure 9A). Next, we evaluated MPO‐DNA levels in our in‐house GC cohort using ELISA. Consistent with our expectation, NETs levels were positively correlated with ANGPTL4 expression and were significantly upregulated in tumor tissues from EBVnGC patients (Figure 9B,C). Collectively, these findings suggest that ANGPTL4 may be involved in NETs formation, although the underlying mechanism remains unclear.

**FIGURE 9:**
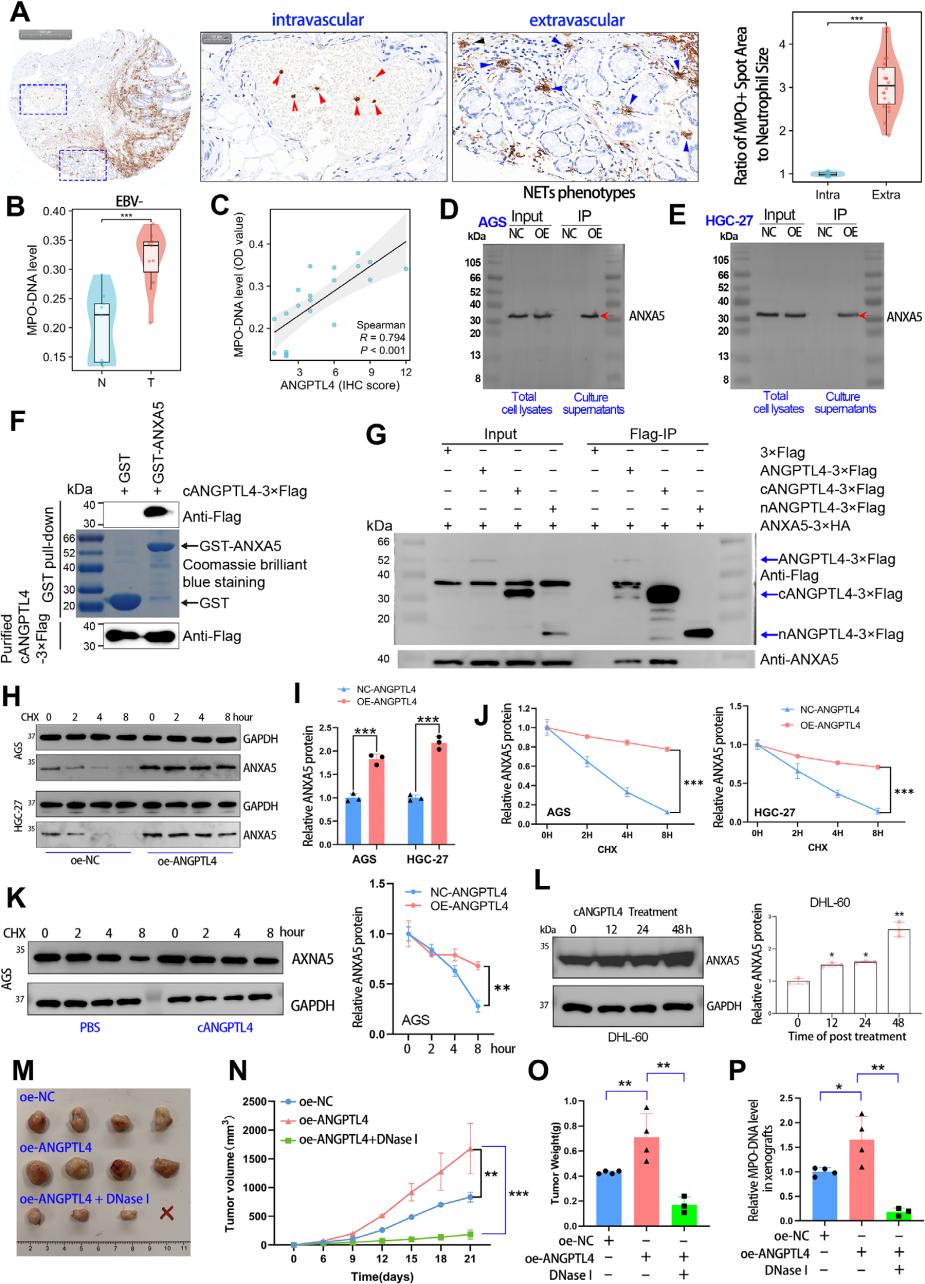
cANGPTL4 interacts with ANXA5 and enhances its stability, thereby promoting the formation of NETs. (A) IHC staining showed that the MPO‐positive extravascular neutrophils was markedly larger than the neutrophil cell body and exhibited a reticular divergent distribution, indicating the formation of NETs. (B, C) The level of NETs was significantly higher in tumor tissues than in paired adjacent normal tissues from EBVnGC patients, and NETs abundance was positively and significantly correlated with ANGPTL4 expression. (D,E) Western blot results confirmed a direct physical interaction between cANGPTL4 and ANXA5 in GC cell lines. (F) GST pull‐down assays confirmed that cANGPTL4 interacts with GST‐tagged ANXA5. (G) Co‐IP analysis using Flag‐tagged ANGPTL4 constructs (full‐length, C‐terminal, and N‐terminal) and ANXA5‐HA showed that ANXA5 binds to both full‐length ANGPTL4 and its C‐terminal fragment, but not the N‐terminal fragment. Western blot results confirmed a direct physical interaction between cANGPTL4 and ANXA5 in GC cell lines. (H–J) The interaction between cANGPTL4 and ANXA5 significantly enhanced the stability of ANXA5 in AGS cells, leading to its significant upregulation in ANXA5‐overexpressing cell lines. (K) cANGPTL4 treatment significantly enhanced the protein stability of ANXA5 in AGS cells. (L) Treatment with cANGPTL4 markedly increased the expression of ANXA5 protein in dHL‐60 neutrophil‐like cells. (M–O) Macroscopic appearance, tumor volume growth curves, and tumor weights in the control, ANGPTL4 overexpression, and ANGPTL4 overexpression + DNase I treatment groups. Overexpression of ANGPTL4 significantly enhanced the growth rate and weight of xenograft tumors in nude mice, and this tumor‐promoting effect was reversed by DNase I treatment. (P) ELISA quantification of intratumoral MPO‐DNA complexes confirmed elevated NETs levels in cANGPTL4‐treated xenograft tumors. *p < 0.05; **p < 0.01; ***p < 0.001.

To further elucidate the biological functions of cANGPTL4, we performed immunoprecipitation coupled with mass spectrometry (IP‐MS) on the culture supernatants to comprehensively identify cANGPTL4‐interacting proteins (Figure S5A). Our IP‐MS assays identified Annexin A5 (ANXA5) as a candidate interacting protein of ANGPTL4 (Figure S5A–C). First, ANXA5 expression was positively correlated with the EMT signaling pathway in GC (Figure S5D). Second, ANXA5 can be expressed in neutrophils and its expression levels are positively correlated with neutrophil infiltration in GC (Figure S5E,F). In addition, clinical analysis showed that ANXA5 expression is significantly upregulated, and its overexpression was significantly associated with malignant progression and poor prognosis in GC (Figure S5G–M). More importantly, Zhang et al. demonstrated that ANXA5 overexpression in neutrophils plays a critical role in inducing NETosis and neutrophil extracellular traps (NETs) release [33].

Thus, we initially validated the interaction between ANXA5 and ANGPTL4 in culture supernatants using western blot analysis (Figure 9D,E). Co‐immunoprecipitation (co‐IP) and GST pull‐down assays confirmed that ANXA5 physically interacts primarily with cANGPTL4 and full‐length ANGPTL4, but not with nANGPTL4 (Figure 9F,G). Moreover, ANGPTL4 overexpression in GC cell lines significantly upregulated ANXA5 protein levels and markedly enhanced its protein stability (Figure 9H–J). Consistently, recombinant human cANGPTL4 protein treatment similarly augmented the stability of the ANXA5 protein (Figure 9K). Given that ANXA5 is also expressed in neutrophils, we further examined the effect of cANGPTL4 treatment on ANXA5 protein levels in dHL‐60 cells, an in vitro induced neutrophil‐like model. Notably, cANGPTL4 treatment significantly enhanced ANXA5 protein levels in dHL‐60 cells in a time‐dependent manner (Figure 9L).

Accumulating evidence has validated that NETs exert oncogenic effects during gastric carcinogenesis and progression [34, 35, 36]. To investigate whether NETs mediate the pro‐tumor function of ANGPTL4, we performed in vivo rescue assays in nude mice (Figure 9M–P). ANGPTL4 overexpression markedly elevated NETs abundance (MPO‐DNA) within xenograft tumors, and the growth‐promoting phenotype induced by ANGPTL4 overexpression was fully rescued by DNase I treatment (Figure 9M–P). Consistently, gene expression correlation analysis showed that ANXA5 and ANGPTL4 were both highly co‐expressed with neutrophil marker genes (S100A8, S100A9, CXCR1/2, CSF3R, and FCGR3B) and NETs formation‐related genes (MPO, PADI4, and ELANE) in GC (Figure S6A,B). Furthermore, ANXA5 expression was significantly upregulated during PMA‐induced NETosis in neutrophils (Figure S6C). And cANGPTL4 treatment significantly induced NETosis in dHL‐60 cells, while ANXA5 knockdown in dHL‐60 cells suppressed NETosis and reversed the pro‐NETosis effect triggered by cANGPTL4 (Figure S6D).

Collectively, these data indicate that ANGPTL4 facilitates the formation and release of NETs via stabilizing ANXA5 protein, thereby driving the progression of GC. In summary, ANGPTL4 not only recruits neutrophils but also participate in neutrophil NETosis and NET release via stabilizing ANXA5 protein.

## 4 Discussion

Epstein‐Barr virus‐associated gastric cancer (EBVaGC) exhibits distinct molecular pathological features, characterized by widespread DNA hypermethylation across the genome [37]. Theoretically, this aberrant epigenetic modification leads to the silencing of relevant genes, including tumor suppressor genes and oncogenes. Notably, the epigenetic silencing of tumor suppressor genes has garnered substantial attention [38]. EBV infection serves as a critical driver in the tumorigenesis and metastasis of EBVaGC by mediating the transcriptional silencing of these tumor suppressor genes [39]. However, an apparent clinical paradox remains: EBVaGC patients generally exhibit superior prognoses and exceptionally high response rates to immune checkpoint inhibitors compared to their EBV‐negative (EBVnGC) counterparts [15]. A retrospective study by Li and colleagues showed that EBVaGC group patients were predominantly male and significantly younger and exhibited significantly higher Ki67 and PD‐L1 expression levels, as well as a lower rate of peritumoral neural invasion [40]. This phenomenon suggests that EBV infection exerts a dual biological effect. While it promotes EBVaGC tumorigenesis by driving tumor suppressor gene inactivation, it may also mediate the transcriptional silencing of specific oncogenes, thereby attenuate tumor malignant progression and metastasis to a certain extent, ultimately conferring a survival advantage to patients. Therefore, it is imperative to comprehensively and deeply examine the global DNA methylation alterations induced by EBV infection, particularly to re‐evaluate the methylation status of oncogene regions and their potential regulatory roles in tumor tumorigenesis and progression.

In this study, we identified *ANGPTL4* as an oncogene subjected to epigenetic silencing by EBV. In EBVaGC, EBV induces DNA hypermethylation at the *ANGPTL4* promoter, leading to its transcriptional silencing. Given that EBVaGC accounts for less than 10% of all gastric cancers, we focused on investigating the functional roles and underlying mechanisms of *ANGPTL4* in EBV‐negative gastric cancer (EBVnGC). In EBVnGC, promoter hypomethylation is associated with a significant upregulation of both *ANGPTL4* transcripts and protein levels in tumor tissues. Furthermore, *ANGPTL4* overexpression is significantly and positively correlated with poor prognosis and distant metastasis in EBVnGC patients. Both in vitro and in vivo experiments confirmed that *ANGPTL4* positively regulates GC cell proliferation, epithelial‐mesenchymal transition (EMT), and metastasis, while suppressing apoptosis. Furthermore, we found that extracellular ANGPTL4 exists primarily in the form of cANGPTL4 in GC. Moreover, treatment with cANGPTL4 alone was sufficient to drive GC cell proliferation, EMT, and metastasis (Figure 8M–O). Collectively, these findings suggest that *ANGPTL4* exerts an oncogenic role in promoting EBVnGC proliferation, EMT, and metastasis through the secretion of cANGPTL4.

Previous studies have reported conflicting findings regarding the biological role of ANGPTL4 in GC. Okochi‐Takada and colleagues demonstrated an antiangiogenic tumor‐suppressive effect of ANGPTL4 [24], whereas Baba et al. showed that ANGPTL4 sustains growth and anoikis resistance in scirrhous GC [41]. Tan et al. further linked cANGPTL4‐dependent tumor invasiveness to integrin α5β1 binding, which was weakened by the T266M variant [25]. In the present study, EBV‐associated promoter hypermethylation helps explain differences in ANGPTL4 expression between EBVaGC and EBVnGC. ANGPTL4 can be secreted by diverse cell populations, and its expression is regulated by cellular context and status. More importantly, most previous studies exploring the biological functions of ANGPTL4 have not adequately distinguished whether the functional form is full‐length ANGPTL4, the C‐terminal fragment cANGPTL4, or the N‐terminal fragment nANGPTL4. For instance, Lauren histological subtype [42], H. pylori‐triggered inflammatory milieu [43], HIF‐1α‐dependent hypoxic induction [41, 44], and LGALS7/Hedgehog signaling crosstalk are known to shape ANGPTL4 function in a cell‐context‐dependent manner; LGALS7 and Hedgehog pathways are reported to switch ANGPTL4 from oncogenic to tumor‐suppressive effects depending on GC cellular backgrounds, and HIF‐1α can transcriptionally upregulate ANGPTL4 under hypoxia in scirrhous gastric carcinoma [45]. Together, these data highlight that ANGPTL4 exerts divergent immune‐modulatory and oncogenic functions, determined by a composite set of factors including EBV epigenetic modification, histological subtype, H. pylori infection status, hypoxia and LGALS7/Hedgehog signaling. Collectively, these lines of evidence indicate that ANGPTL4 exerts distinct immunoregulatory and pro‐tumor effects, depending on its functional isoform and the regulatory mechanisms governing its expression, including EBV‐mediated epigenetic modification, histological subtype, H. pylori infection, hypoxia, LGALS7/Hedgehog signaling, and other multiple contributing factors.

Another novelty of this study lies in elucidating the critical role of the secreted protein cANGPTL4 within the tumor microenvironment (TME). In GC, both the infiltration level of neutrophils and the levels of secreted ANGPTL4 (cANGPTL4) in the serum are significantly elevated (Figure 6C and Figure 8G). Moreover, we noticed that *ANGPTL4* expression levels are significantly and positively correlated with neutrophil infiltration, accompanied by spatial colocalization. Specifically, regions with high *ANGPTL4* expression exhibit a marked enrichment of neutrophils (Figure 6E–G). Prognostic analysis revealed that GC patients with both high *ANGPTL4* expression and high neutrophil infiltration have the poorest prognosis (Figure 6H–J). Mechanistic investigations demonstrated that *ANGPTL4* promotes neutrophil recruitment by activating the NF‐κB signaling pathway to upregulate the chemokine CXCL8 (Figure 7).

Neutrophils play a pivotal role in shaping the immunosuppressive TME and promoting tumor immune evasion [46]. Studies have demonstrated that neutrophils can directly inhibit the activation of effector cytotoxic cells (such as T cells and NK cells) and induce their functional exhaustion by releasing various immunosuppressive mediators [47, 48]. Concurrently, the overexpression of immune checkpoint molecules on the surface of neutrophils negatively regulates T cell function, thereby mediating tumor resistance to immunotherapy [49]. Furthermore, neutrophils can also promote tumor immune evasion by releasing neutrophil extracellular traps (NETs) via NETosis [50, 51]. More importantly, neutrophils can induce DNA damage in tumor cells by generating reactive oxygen species (ROS), driving the loss or mutation of tumor antigens. Alternatively, they can regulate the expression of epigenetic modifiers within tumor cells through the secretion of exosomes [52].

In this study, our findings suggest that ANGPTL4 is involved in the remodeling of an immunosuppressive tumor microenvironment by recruiting tumor‐associated neutrophils (TANs) and inducing NETosis and NET formation. Therefore, this may explain why patients with EBV‐positive gastric cancer (EBVaGC) exhibit superior immunotherapy responses, which can be attributed to EBV‐mediated epigenetic silencing of ANGPTL4. Nevertheless, a limitation of this study is the use of nude mice, which lack functional T cells and therefore do not allow a complete assessment of the adaptive immune response. Consequently, our findings may not fully reflect the immune context of immunocompetent hosts. Future studies using immunocompetent animal models will be needed to further validate these results and to better characterize the role of adaptive immunity.

In the present study, we identified ANXA5 as a novel protein that physically interacts with secreted ANGPTL4. Mechanistically, secreted ANGPTL4 enhances the protein stability of ANXA5 through direct physical binding. Previous reports have indicated that ANXA5 upregulation promotes neutrophil NETosis and the formation of NETs [33]. Consistent with these findings, our study demonstrates that cANGPTL4 promotes NET formation in GC tissues by enhancing ANXA5 protein stability (Figure 9K–P), thereby driving stomach cancer progression.

A tumor is not merely a homogeneous mass of malignant cells, but rather a complex ecosystem composed of diverse cell types, extracellular matrix, and biomolecules [53]. Fundamentally, TME is a highly heterogeneous and dynamically plastic system whose continuous evolution is modulated by multiple factors, including the patient's age, systemic inflammatory status, and external environment [54]. Although immune checkpoint inhibitors (ICIs) have achieved breakthroughs in various solid tumors, their clinical benefits in GC remain suboptimal [55]. Fundamentally, GC is typically classified as a prototypical “cold tumor,” characterized by a scarcity of effector T‐cell infiltration within the TME and the presence of a prominent immunosuppressive barrier [56]. Furthermore, GC is generally characterized by a low tumor mutational burden (TMB) and low PD‐L1 expression levels, which hinder ICIs from effectively activating specific anti‐tumor immune responses [57]. This lack of immunogenicity directly limits the clinical efficacy of ICI monotherapy in GC. Therefore, how to remodel the TME and transform “cold tumors” into “hot tumors” has emerged as a critical scientific question for improving the response rate of immunotherapy in GC [54].

Neutrophil infiltration is instrumental in shaping the immunosuppressive microenvironment of GC (Figure 6A), with secreted proteins serving as key mediators in the formation of immunosuppressive niches. Here, we demonstrate that secreted ANGPTL4 (cANGPTL4) drives GC progression via a dual mechanism (Figure 10). On one hand, cANGPTL4 directly promotes GC cell proliferation, EMT, and metastasis. On the other hand, cANGPTL4 activates the NF‐κB/CXCL8 signaling pathway to promote TANs infiltration into the peritumoral region, and directly binds to neutrophil‐derived ANXA5 to induce NET release. In summary, ANGPTL4 serves as a potential therapeutic target for EBVnGC. Targeting ANGPTL4 may achieve a “two birds with one stone” anti‐tumor effect: not only directly inhibiting proliferation, EMT, and metastasis in GC, but also remodeling the immunosuppressive microenvironment by reducing TAN infiltration and NETs release.

**FIGURE 10:**
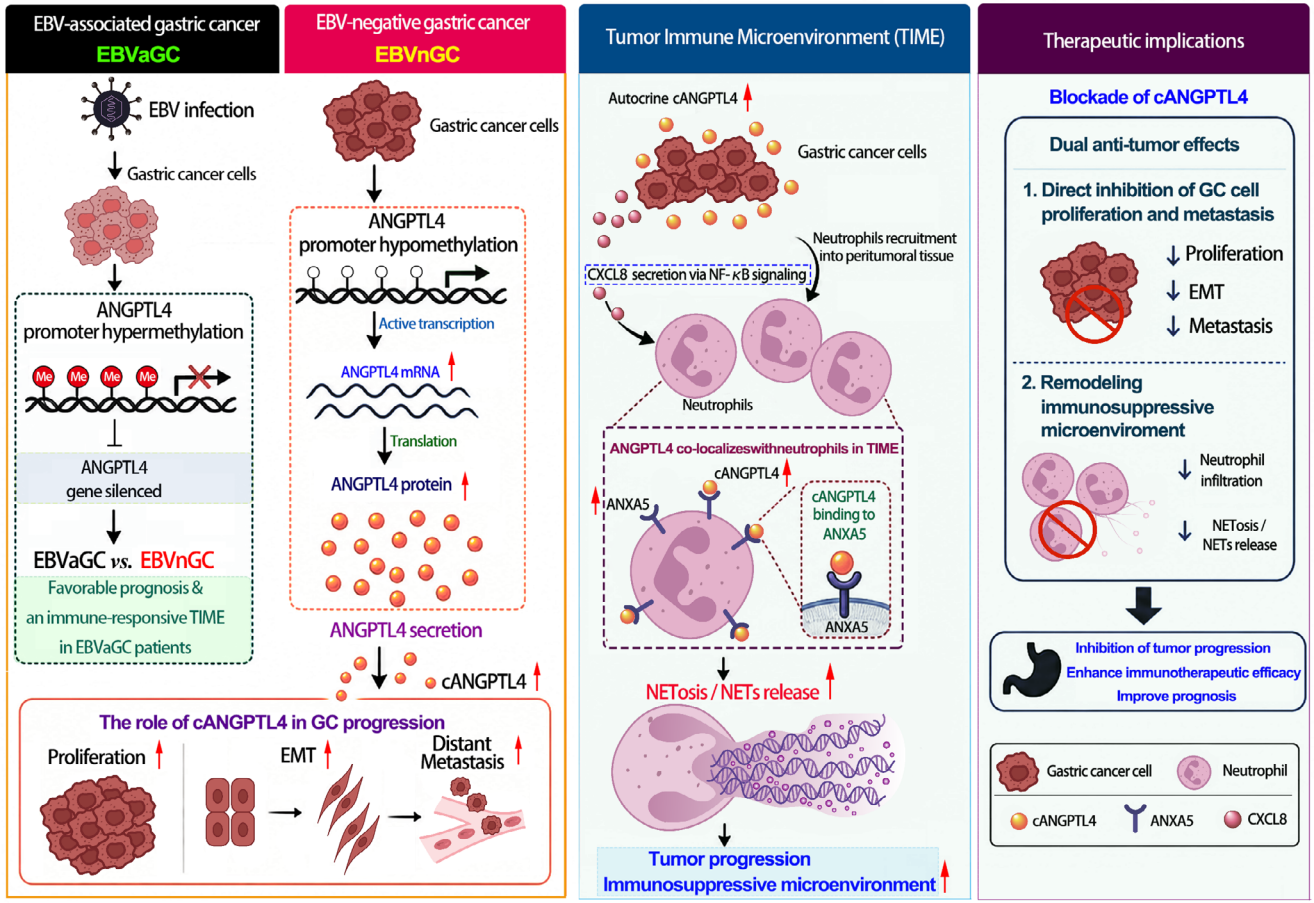
Schematic diagram illustrating the working mechanism of ANGPTL4 in EBVnGC. In EBVaGC, ANGPTL4 expression is silenced due to EBV infection‐induced promoter hypermethylation. In EBVnGC, driven by promoter hypomethylation, ANGPTL4 is overexpressed and closely associated with unfavorable prognosis and distant metastasis in patients. Its secreted C‐terminal fragment (cANGPTL4), acting as the dominant functional isoform, not only directly drives tumor proliferation, EMT, and metastasis, but also recruits tumor‐infiltrating neutrophils via autocrine activation of the NF‐κB/CXCL8 axis and induces NETs release through interaction with and stabilization of ANXA5, collectively establishing an immunosuppressive microenvironment. Therefore, targeting ANGPTL4 could not only directly suppress GC cell proliferation, EMT, and metastasis, but also remodel the immunosuppressive microenvironment by attenuating neutrophil infiltration, thereby exerting a dual anti‐tumor therapeutic efficacy.

## Author Contributions

**Shanshan Qin**, **Dandan Li**, **Weidong Leng**: Writing‐ Original draft preparation, Funding, Writing‐ Reviewing and Editing. Conceptualization. **Dandan Li**, **Xinqi Li**, **Xiangyin Luo**, **Zeng Zhou**, **Jiayi Wang**, **Yanyan Chen**, **Xiangyin Luo**, **Qi Wang**, **Yiwei Zha**, and **Lingyun Xia**: Investigation, Methodology.

## Funding

This study was supported by grants from the National Natural Science Foundation of China (82673731, 82273451, 32402391); Hubei Provincial Natural Science Foundation (2025BCB063); the Top Young Talents of Medicine in Hubei Province (2024‐2027) and the Project of the Innovation and Development Joint Fund of the Hubei Provincial Natural Science Foundation (2025AFD183, 2026AFC0277, and 2025AFD200), the Biomedical Research Foundation for PI of Hubei University of Medicine HBMUPI202504, the Advantages Discipline Group (Medicine) Project in Higher Education of Hubei Province (2021‐2025, 2025XKQY21), and the Faculty Development Grants from Hubei University of Medicine (2024QDJZR009 to ZZ, 2025QDJZR14 to CYY).

## Ethics Statement

The study was approved by the Ethics Committee of Hubei University of Medicine (2026‐104) in accordance with the Declaration of Helsinki. All patient materials were collected after obtaining informed consent.

## Conflicts of Interest

The authors declare no conflicts of interest.

## Supporting information




**Supporting File**: advs78595‐sup‐0001‐SuppMat.docx.

## Data Availability

The data that support the findings of this study are openly available in GEO datasets at https://www.ncbi.nlm.nih.gov/gds, reference number GSE336542, GSE336544, and GSE338826.
