# Supplementary material for: Epigenetic Activation of ANGPTL4 Drives EBV‐Negative Gastric Cancer via Neutrophil Recruitment and NET Release via Stabilizing ANXA5
Source: Adv Sci (Weinh). 2026 Nov 5:e78595. Online ahead of print. doi: 10.1002/advs.78595 (PMC13735440; doi:10.1002/advs.78595)
Supplement: Supplementary file 1 [file ADVS-9999-e78595-s001.docx]

**Supplementary File**

**Figure S1-S6**

**Figure S1** Spatial transcriptomics analysis revealed the distribution characteristics of different cell types within gastric cancer tissues.

**Figure S2** IHC analysis demonstrated a marked enrichment of tumor-infiltrating neutrophils (marked by MPO) in the peritumoral stroma region.

**Figure S3** The activation of EMT signaling pathways by ANGPTL4 is dependent on its secretion. **(A)** Schematic diagram of the conditioned medium (CM) treatment assay. Wild-type HGC-27 cells were treated with oe-NC_CM or oe-ANGPTL4_CM for 72 hours, followed by RNA-seq analysis (GSE338826). **(B, C)** RNA-seq analysis revealed that treatment with oe-ANGPTL4_CM significantly activated the expression of EMT-related transcription factors in wild-type HGC-27 cells.

**Figure S4** cANGPTL4 treatment significantly promoted the growth rate and tumor volume in nude mouse xenograft models. **(A)** Schematic diagram of the nude mouse subcutaneous xenograft model evaluating the effect of cANGPTL4 on tumor growth. **(B-D)** Macroscopic appearance, tumor weights, and tumor volume growth curves in the control, cANGPTL4 treatment groups. cANGPTL4 treatment significantly enhanced the growth rate and weight of xenograft tumors in nude mice.

**Figure S5** Expression Characteristics and Clinical Significance of ANXA5 in GC. (**A**) Schematic diagram illustrating the identification of cANGPTL4-interacting proteins using IP-MS. (**B**) Potential binding proteins of cANGPTL4 in the IP samples were identified by mass spectrometry, following SDS-PAGE separation and Coomassie brilliant blue staining. (**C**) Venn diagram analysis of the IP-MS results revealed that a total of 472 proteins were specifically present in the IP-OE samples, including ANXA5. (**D**) In the TCGA-STAD cohort, ANXA5 expression was significantly positively correlated with the EMT signaling pathway in GC. (**E**) Based on the HPA database, we observed that ANXA5 can be expressed in neutrophils. (**F**) In the TCGA-STAD cohort, the expression level of ANXA5 was significantly positively correlated with neutrophil infiltration. (**G**) In the TCGA-STAD cohort, ANXA5 expression was significantly upregulated in GC. (**H**) In the TCGA-STAD cohort, ANXA5 expression positively correlated with pathological T stages in GC. (**I**) Expression levels of ANXA5 in tissues of patients with different pathological stages in TCGA-STAD cohort. (**J, K**) In the TCGA-STAD cohort, patients with high ANXA5 expression exhibited significantly shorter overall survival (OS) and progression-free survival (PFS) times. (**L, M**) ANXA5 expression levels were significantly higher in gastric cancer patients who experienced overall survival (OS) and disease-specific survival (DSS) events.

**Figure S6** ANXA5 expression is positively correlated with neutrophil extracellular traps (NETs) and is significantly upregulated during NETosis. (**A, B**) In the TCGA-STAD cohort, ANXA5 or ANGPTL4 expression exhibits a positive correlation with neutrophil markers and NETs formation-related genes. (**C**) ANXA5 expression in neutrophils is markedly upregulated in the PMA-induced NETosis. (**D**) cANGPTL4 treatment markedly promotes NETosis in dHL‑60 cells. ANXA5 knockdown in dHL‑60 cells suppresses NETosis per se. Moreover, ANXA5 knockdown reverses the pro‑NETosis effect of cANGPTL4 in neutrophils.
